# Optimization and characterization of silver nanoparticle-modified luffa for the adsorption of ketoprofen and reactive yellow 15 from aqueous solutions

**DOI:** 10.1038/s41598-024-54790-7

**Published:** 2024-02-22

**Authors:** Soheil Tavassoli, Setareh Cheraghi, Pardis Etemadifar, Afsaneh Mollahosseini, Shirin joodaki, Niloofar Sedighi

**Affiliations:** Research Laboratory of Spectroscopy and Micro and Nano Extraction, Department of Chemistry, Iran University of Science and Technology (IUST), Narmak, Tehran, Iran

**Keywords:** Adsorption, Ketoprofen, Dye, Luffa, Nanoparticles, Pollution remediation, Chemical engineering

## Abstract

In the current work, luffa was modified with silver nanoparticles to prepare LF/AgNPs adsorbent for the elimination of ketoprofen and reactive yellow 15 (RY15) from aqueous media. Various characterization techniques, including FT-IR, XRD, BET, and SEM–EDS analysis, were employed to confirm the successful modification of LF/AgNPs. Several key parameters such as contact time, adsorbent dosage, concentration, pH, and agitation technique were fine-tuned to optimize the adsorption process. Ketoprofen removal was found to be most effective in weakly acidic conditions (pH = 5), while reactive yellow 15 adsorption was enhanced in an acidic environment (pH = 2). At 298 K, the highest adsorption capacities reached 56.88 mg/g for ketoprofen and 97.76 mg/g for reactive yellow 15. In both scenarios involving the elimination of ketoprofen and RY15, the Temkin isotherm exhibits higher R^2^ values, specifically 0.997 for ketoprofen and 0.963 for RY15, demonstrating a strong correlation with the observed adsorption data. Additionally, the kinetics of ketoprofen adsorption were best described by the Pseudo-first order model (R^2^ = 0.989), whereas the Pseudo-second order model provided the most accurate fit for reactive yellow 15 adsorption (R^2^ = 0.997). Importantly, the LF/AgNPs adsorbent displayed consistent performance over five consecutive reuse cycles, affirming its stability and efficacy in removing both contaminants. These findings underscore the exceptional potential of LF/AgNPs as a reliable adsorbent for the removal of reactive yellow 15 and ketoprofen from aqueous solutions.

## Introduction

Water is an essential element of ecosystem. The existence of pharmaceutical products and chemical dyes in water and wastewater is toxic and carcinogenic, posing a threat to all living organisms on the trophic chain, including human health. As soon as they released in water, it becomes unsafe for drinking and so resulting numeral health problems. Consequently, the elimination of these contaminants is imperative. While water is contaminated, several consequences can occur, such as temperature and color changing, undesired acidity or alkalinity, unpleasant odor, and turbidity caused by organic or inorganic solids. Moreover, the generated wastewater of various industries such as food production, leather processing, paper production, printing, paints, and cosmetics, which also contain dyes, pose a significant threat to the environment by being discharged into fresh waters^1–10^.

Azo colors constitute around 70% of the 800,000 tons of colors delivered globally each year, making them the most used and hazardous dyes in the textile industry. Due to their aromatic complex nature, azo dyes resist traditional wastewater handling methods, posing environmental challenges. Reactive dyes, including azo, anthraquinone, oxazine, and phthalocyanine groups, are favored for their enduring color in textiles. However, the toxicity of dyestuffs, especially azo colors, is a growing concern, requiring innovative and cost-effective methods for their removal from material wastewater. This is vital due to the high resistance of azo colors to sunlight degradation, posing a substantial environmental risk^11–17^.

Pharmaceuticals, including the widely used non-prescription anti-inflammatory medication ketoprofen (2-(3-benzoylphenyl) propionic acid), have been found in groundwater, surface water, and streams, as well as in sludge, soil, and sediment samples. The inefficiency of treatment plants contributes to this presence. Ketoprofen, known for its affordability, is commonly prescribed for muscle and joint pain, arthritis, and inflammation, but prolonged or excessive use can lead to adverse health effects, including gastrointestinal problems. Despite the lack of specific legislation, increasing environmental and health concerns have prompted the scientific community to improve methods for the elimination of pharmaceutical compounds like ketoprofen^18–25^.

Therefore, there is a pressing need for an effective system to eliminate these compounds from aquatic environments. Various methods, including separation membrane, chemical oxidation, and biodegradation, have been utilized for organic compound removal. However, these approaches often produce secondary pollutants or involve time-consuming processes. The adsorption process, employing affordable and readily available adsorbents, stands out as one of the most efficient, straightforward, and cost-effective methods for removing dyes and drugs^26–35^. As the effectiveness of adsorption is closely tied to the quality and cost efficiency of the adsorbent, diverse materials have undergone scrutiny in recent years. The application of luffa in adsorption processes has garnered significant acclaim among researchers^36–41^. Luffa is composed of three main components: cellulose (60%), hemicelluloses (30%), and lignin (10%), classifying as a lignocellulosic material. Its fibrous nature, large surface area, impressive mechanical strength, low cost, light weight, and renewable origin are its most notable advantages.^42–44^.

This investigation signifies an innovative effort as it introduces a novel modification process applied to luffa for the first time, involving silver nanoparticles. This transformation makes it a highly effective adsorbent for removing both ketoprofen and reactive yellow 15 from water. Raw luffa inherently possesses a limited capacity for adsorbing contaminants. To overcome this limitation, luffa was modified with silver nanoparticles. Silver nanoparticles are recognized for their significant adsorption capacity for various chemical species, including methylene blue, agrochemicals, and organic compounds like quinolones, pyrene, and ketones. Moreover, they have been employed for the adsorption of inorganic compounds^45–50^. Silver nanoparticles meticulously prepared via the chemical oxidation method. The selection of the chemical oxidation method was driven by its effectiveness in surface functionalization, increased surface area, enhanced adsorption affinity, and its alignment with eco-friendly practices^51–53^. This resulted in the creation of a novel nanocomposite, denoted as luffa-silver nanoparticles (LF/AgNPs). Essentially, luffa served as a supportive matrix for the silver nanoparticles, enhancing its adsorptive capabilities. The LF/AgNPs displayed remarkable potential as an adsorbent and exhibited substantial removal rates. Notably, LF/AgNPs offered a cost-effective, eco-friendly, and non-toxic solution for the elimination of ketoprofen and reactive yellow 15. The study precisely explored various crucial parameters, including pH, contact time, concentration, adsorbent dosage, and agitation technique to optimize the adsorption process. Furthermore, the research delved into mathematical modeling, precisely assessing the kinetics and adsorption isotherms, providing valuable insights into the intricate adsorption mechanisms at play.

## Materials and chemicals

### Materials and equipment

Ketoprofen and RY15 were obtained from Temadkala Pharmaceutical Company (IRAN) with a purity of more than 99%. The specific chemicals used in this study including polyvinyl pyrrolidone (1000 MV), ethane-1,2-diol (99.8%), silver nitrate (99.99%), 3-aminopropyl triethoxy silane (APTES) (99%), 2-hydroxybenzaldehyde (99%), ethanol (EtOH) (96%), methyl alcohol (99.99%), hydrochloric acid (37%), and sodium hydroxide were purchased from Merck Company.

### Synthesis of luffa powders

The luffa was purchase from a store located in Isfahan. The luffa was reduced in size by being fragmented and purified with distilled water to eliminate any foreign matters that could dissolve into the water. Subsequently, it was dehydrated in an oven set at 40 °C for 4 h. After that, the reduced fragments of luffa were ground for 25 min using a ball mill operating at 20 Hz, resulting in white luffa powder.

### Applying silver nanoparticles to the surface of luffa for modifying

APTES and 2-hydroxybenzaldehyde were utilized as connectors to surge the quantity of Ag on the surface of luffa through silver nanoparticle modification^54^. The first step involved refluxing 1 g of luffa powder with 70 mmol APTES and 25 ml of EtOH for 48 h, resulting in a precipitate labeled A. The solid substance obtained was subjected to filtration and multiple washing procedures with DI water and EtOH and dried at 40 °C for 4 h. Next, substance A in solid form was refluxed with 10 mmol 2-hydroxybenzaldehyde and 25 ml EtOH for 24 h, resulting in another precipitate named B. This collected solid was subjected to filtration and multiple rounds of washing with DI water and ethanol, and dried at 40 °C.

**Figure 1:**
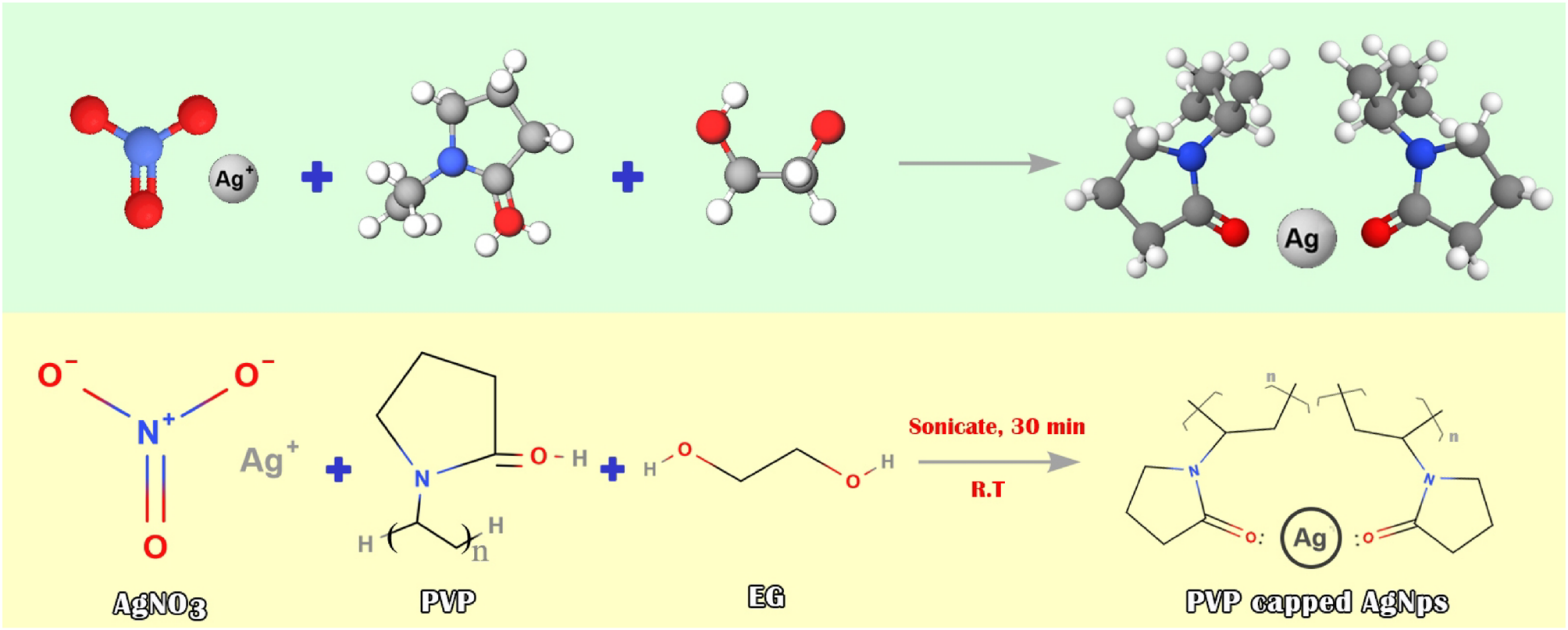
The potential interaction of PVP and AgNO_3_ during the formation of AgNPs.

The chemical reduction approach was employed to synthesize silver nanoparticles in the third step^55^. AgNO_3_ was utilized as a precursor, and polyvinylpyrrolidone (PVP) and ethylene glycol (EG) were utilized as stabilizer and reducing agent, respectively. To dissolve PVP in EG, a flask was loaded with 40 ml of Ethylene glycol and 4 g of PVP and placed in an ultrasonic bath. The potential interaction that may occur between PVP and silver nitrate in the process of AgNPs formation is illustrated in Fig. 1.

A reddish-brown solution, called Solution C, was produced by gradually adding a solution to 2 mmol of silver nitrate in a container that was protected by aluminum foil. The container was then put in an ultrasonic bath at 25 °C for duration of 30 min until the reaction was completed.

**Figure 2:**
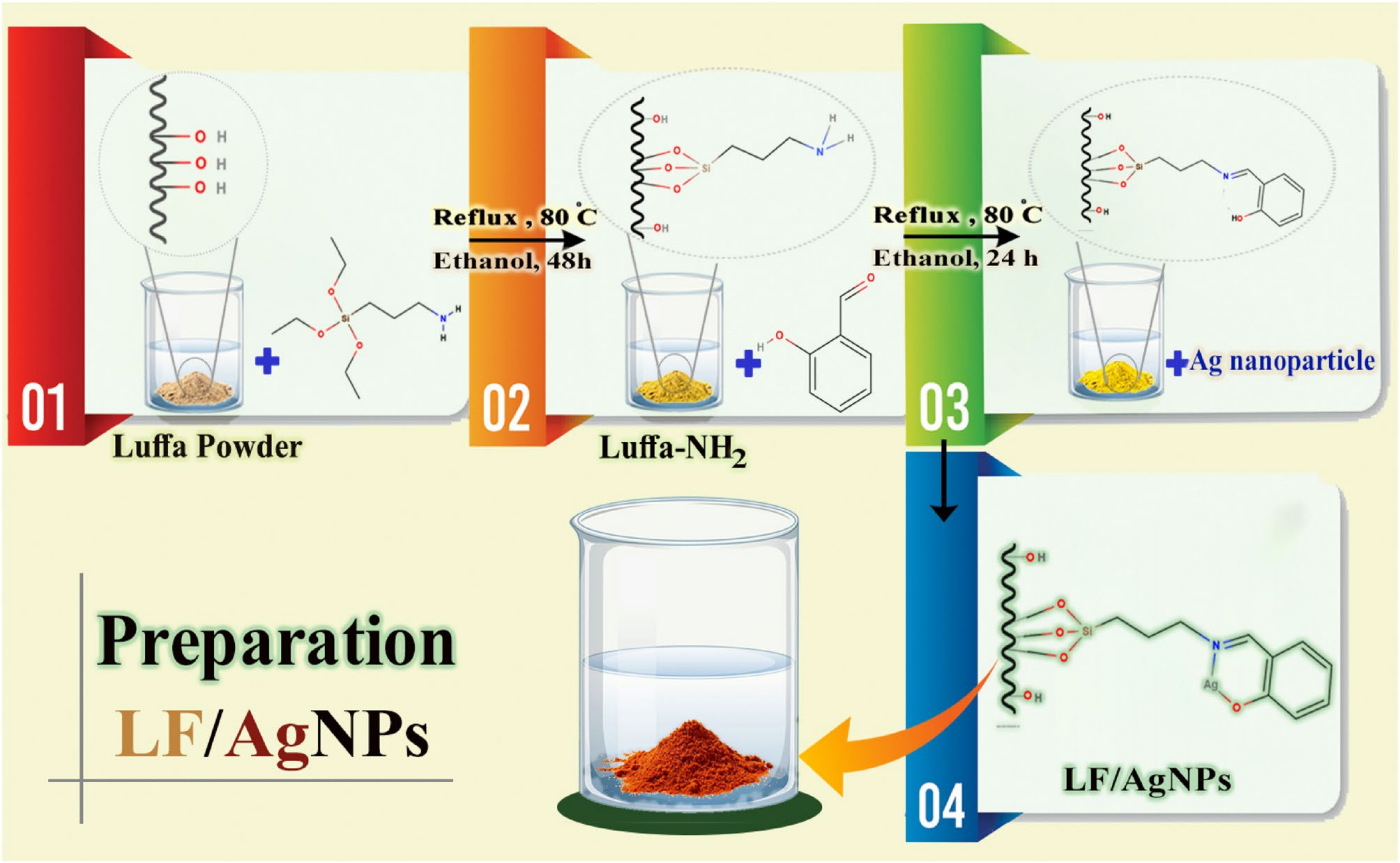
The preparation process of LF/AgNPs.

To modify the surface of the luffa by AgNPs, the magnetic stirrer was used to stir precipitate B and solution C for a duration of 48 h in a dark area. Once the reaction was complete, the brown collected solid was subjected to filtration and multiple rounds of washing with distilled water and EtOH, and subsequently, it was dried for 1 h at a temperature of 40 °C. Figure 2 illustrates the different steps involved in the preparation of the LF/AgNPs adsorbent.

### Experimental method

The adsorption experiments were carried out in a 10 mL falcon tube under consistent agitation using digital vortex mixers at ambient temperature. Investigations were carried out under optimum contact time, contaminant concentration, pH, and adsorbent dosage by one-at-a-time method. A solution of HCl/NaOH with a concentration of 0.1 M was utilized to adjust the pH. To perform the adsorption of ketoprofen and RY15 onto LF/AgNPs, a 10 mL solution of the contaminant was blended with the adsorbent. Following the conclusion of the adsorption process, the solution underwent centrifugation at 5000 rpm for 10 min to separate the LF/AgNPs. The residual concentration of contaminants was then determined spectrophotometrically. The following equations show the calculation methods for ketoprofen and RY15 adsorbed at equilibrium q_e_ (mg/g), and the removal rate:

1 $${{\text{q}}}_{{\text{e}}}=\frac{\left({{\text{C}}}_{0}-{{\text{C}}}_{{\text{e}}}\right)\times {\text{V}}}{{\text{m}}}$$

2 $$\mathrm{Removal efficiency }(\mathrm{\%})=\frac{{{\text{C}}}_{0}-{{\text{C}}}_{{\text{e}}}}{{{\text{C}}}_{0}}\times 100$$

${{\text{C}}}_{0}$ (mg/L) is the starting concentration, ${{\text{C}}}_{{\text{e}}}$ (mg/L) is the equilibrium concentration of contaminant, m (mg) is the adsorbent dosage added to the solution, and V (ml) is the volume of the solution^56,57^.

### Characterization

To study the morphological characteristics and perform elemental analysis of the adsorbent, scanning electron microscopy (SEM) coupled with energy dispersive X-ray (EDAX) analysis was employed. The analysis was carried out using the Te-scan, MIRA III equipment from the Czech Republic. To determine the functional group present in the adsorbents, Fourier Transform Infrared spectrometry (FT-IR) was conducted using a Thermo AVATAR IR spectrophotometer, with a spectral range of 4000–400 cm^−1^. Brunauer–Emmett–Teller (BET) technique was used to obtain the porous structural data for luffa sponge, powdered luffa, and LF/AgNPs. The concentration variations in the solutions were determined using a UV–Visible spectrophotometer (T80 + , PG, UK). Additionally, X-ray diffraction (XRD) patterns were obtained using a diffractometer (Bourevestnik, DRON-8) equipped with Cu K_α_ radiation and operated at 40 kV and 40 mA, over a range (2θ) of 10°–80°.

## Results and discussion

### Characterization

**Figure 3:**
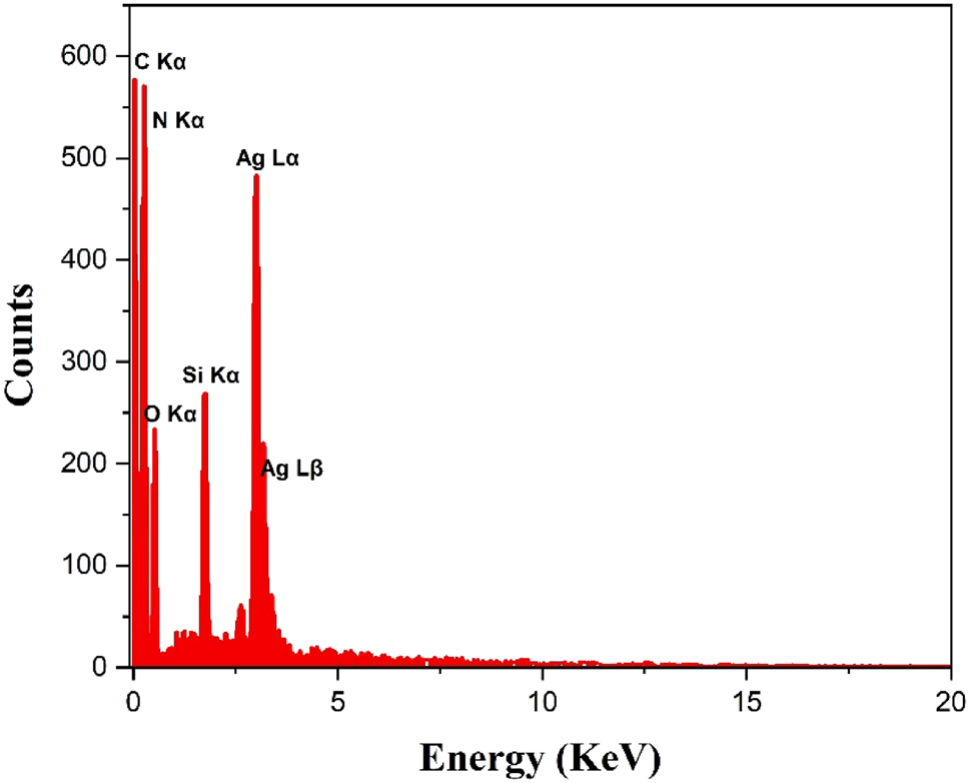
EDAX spectra of LF/AgNPs.

As shown in Fig. 3, EDAX analysis is employed to exhibit the elements present in the LF/AgNPs structure. In addition, the luffa contains the 2-hydroxybenzaldehyde linker, which plays a more significant role compared to other components. The detection of silicon and nitrogen peaks in the analysis can be indicative of the presence of the 3-aminopropyl triethoxysilane linker. To summarize, the appearance of a silver peak in the analysis may show the presence of silver particles on the surface of the LF/AgNPs adsorbent. Moreover, it is evident that the element with the highest distribution is carbon. This can be attributed to the presence of carbon in both the luffa structure and the salicylic aldehyde linker structure.

**Figure 4:**
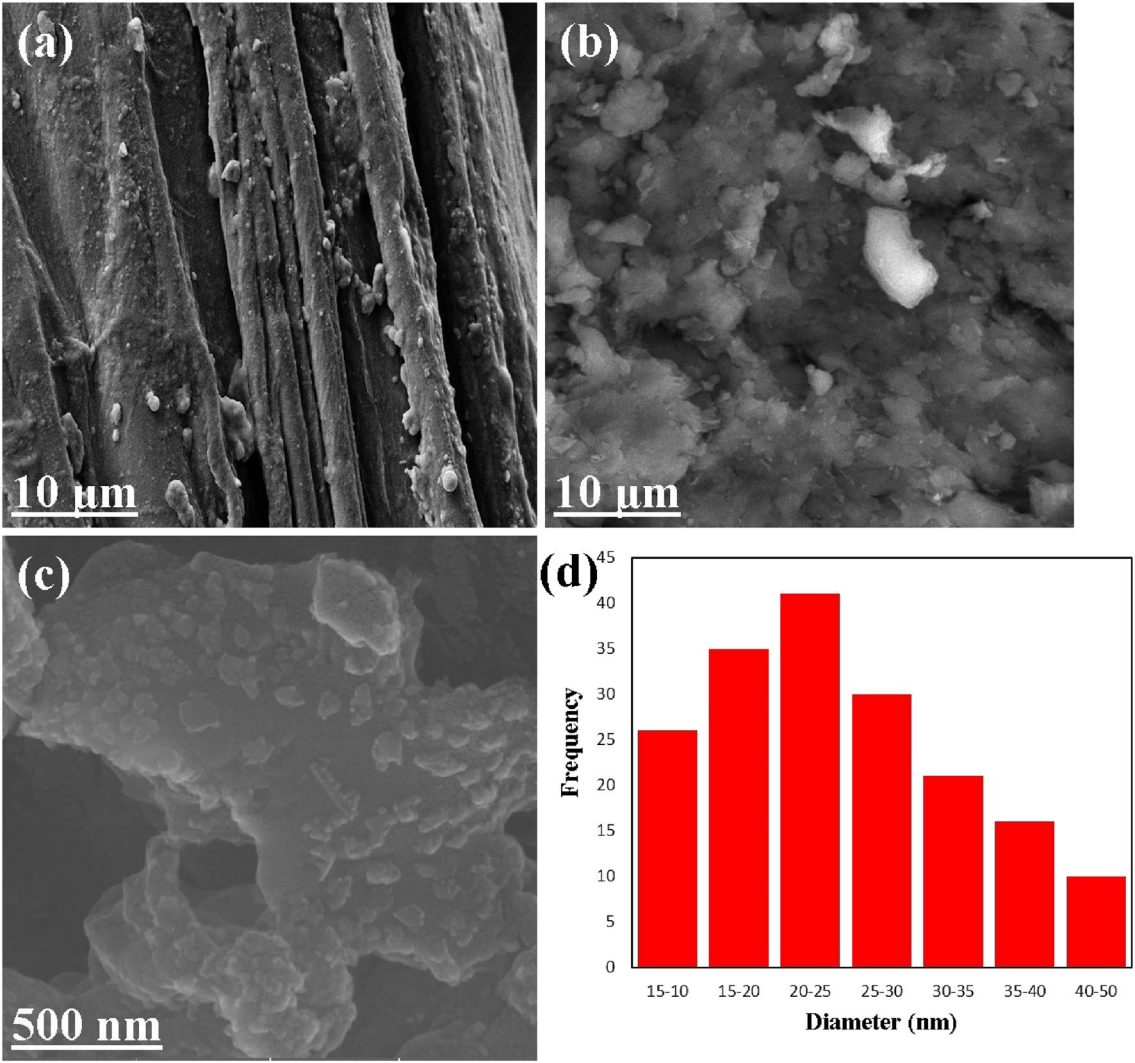
SEM images of (**a**) luffa sponge, (**b**) luffa powder, (**c**) LF/AgNPs, (**d**) size distribution of silver nanoparticles on the LF/AgNPs adsorbent.

Figure 4a depicts the structure of a luffa sponge. The SEM image displays that unmodified luffa has a fibrous structure with varying fiber diameters. However, as seen in Figs. 4b, the fibrous structure is lost in the luffa powders. Figures 4c exhibit pictures of the LF/AgNPs structure, in which the luffa surface is covered with silver nanoparticles having a spherical shape, and their arrangement can be clearly observed. On the LF/AgNPs substrate, Fig. 4d illustrates the size distribution of silver nanoparticles, indicating an average size ranging from 20 to 25 nm.

**Figure 5:**
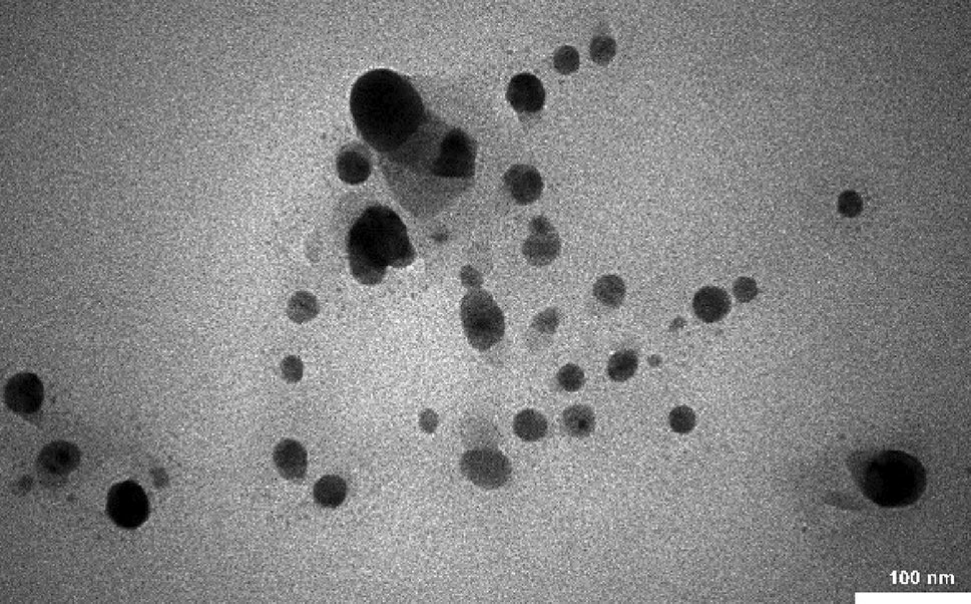
TEM image of LF/AgNPs.

The TEM image of LF/AgNPs offers a detailed glimpse into the structure of silver nanoparticles, as depicted in Fig. 5. The image distinctly illustrates the uniform dispersion of silver nanoparticles within the luffa framework, devoid of any agglomeration. Moreover, the image reveals that the silver nanoparticles assume a spherical form.

Table 1 presents the porous structural data obtained from BET analysis for luffa sponge, powdered luffa, and LF/AgNPs. The luffa sponge exhibits a limited surface area, but after ball-milling, both surface area and pore volume increase. Ball-milling disrupts the fibrous structures of luffa, exposing more surfaces and opening its pores. The surface area of LF/AgNPs is comparable to powdered luffa, but the pore volume is reduced, possibly due to the dispersion of nanoparticles within the pores. While the presence of silver nanoparticles decreases porosity, it enhances active adsorbent sites for the elimination of contaminants.

**Table 1:** The porous structural data obtained from BET analysis.

Sample	Surface area (m^2^/g)	Total pore volume (cm^3^/g)
Luffa sponge	1.440	1.754 × 10^–3^
Powdered luffa	16.531	1.279 × 10^–2^
LF/AgNPs	16.852	6.512 × 10^–3^

**Figure 6:**
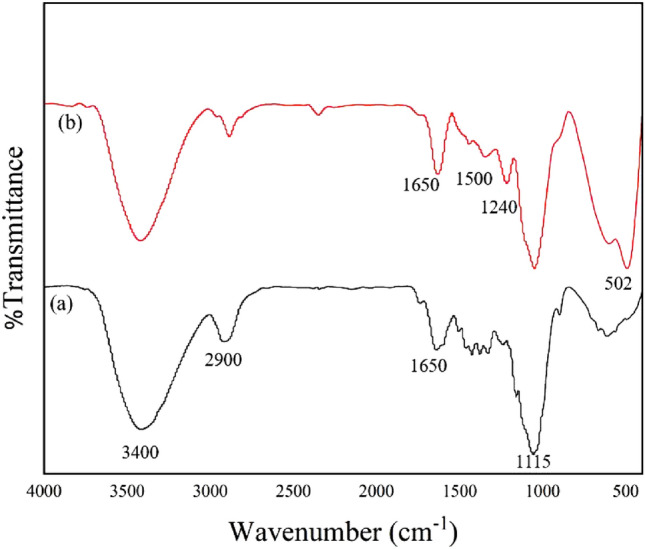
FT-IR spectra of (**a**) raw luffa and (**b**) LF/AgNPs.

FT-IR analysis was conducted to confirm the process of modifying the surface of luffa by using AgNPs. Figure 6a depicts the raw luffa spectrum, and the peak observed around 3400 cm^−1^ is the stretching vibrations of OH– groups on the surface of luffa. Additionally, the peak observed at approximately 2900 cm^−1^ is associated with both symmetric and asymmetric stretching vibrations of C–H bonds. The peak observed around 1650 cm^−1^ is the vibrations of C=C and C=O bonds. The stretching vibration peaks observed at approximately 1055 cm^−1^ and 1115 cm^−1^ suggest the presence of C–OR and anhydroglucose in cellulose, as reported in previous studies^4,42,58^. Figure 6b presents the spectrum of LF/AgNPs which demonstrates the investigation of the binding of 4-aminopropyl triethoxysilane molecules on the surface of luffa. The bending and stretching vibration peaks of the N–H bond were not visible in the spectrum due to the overlapping of the peaks at 1650 cm^−1^ and 3400 cm^−1^. However, it can be observed that the intensity of these peaks has increased^59^. The presence of a Si–O–C peak observed at approximately 1240 cm^−1^, along with the aforementioned peaks, provides evidence of the attachment of an amino linker to the luffa structure, as reported in previous studies^60^. Moreover, the C=N peak observed at 1634 cm^−1^ provides evidence of the binding of salicylic aldehyde to 4-aminopropyl triethoxysilane, which forms an imine group^61^. The vibrational peaks associated with the stretching of C=C bonds in the aromatic ring of salicylic aldehyde are visible at approximately 1634 cm^−1^ and 756 cm^−1^. It is worth noting that the peak observed at 1634 cm^−1^ has overlapped with the previous peaks, and its intensity has increased^62^. Peaks observed in the range of 1400–1500 cm^−1^ are associated with the vibration of the –N–C– group. It should be noted that the stretching peak at 1650 cm^−1^, which belongs to the C=O group of polyvinyl pyrrolidone, is observed to overlap with the previous peaks. Additionally, it is observed that the peak at 502 cm^−1^ corresponds to the vibration of the Ag–O bond^42,63,64^.

**Figure 7:**
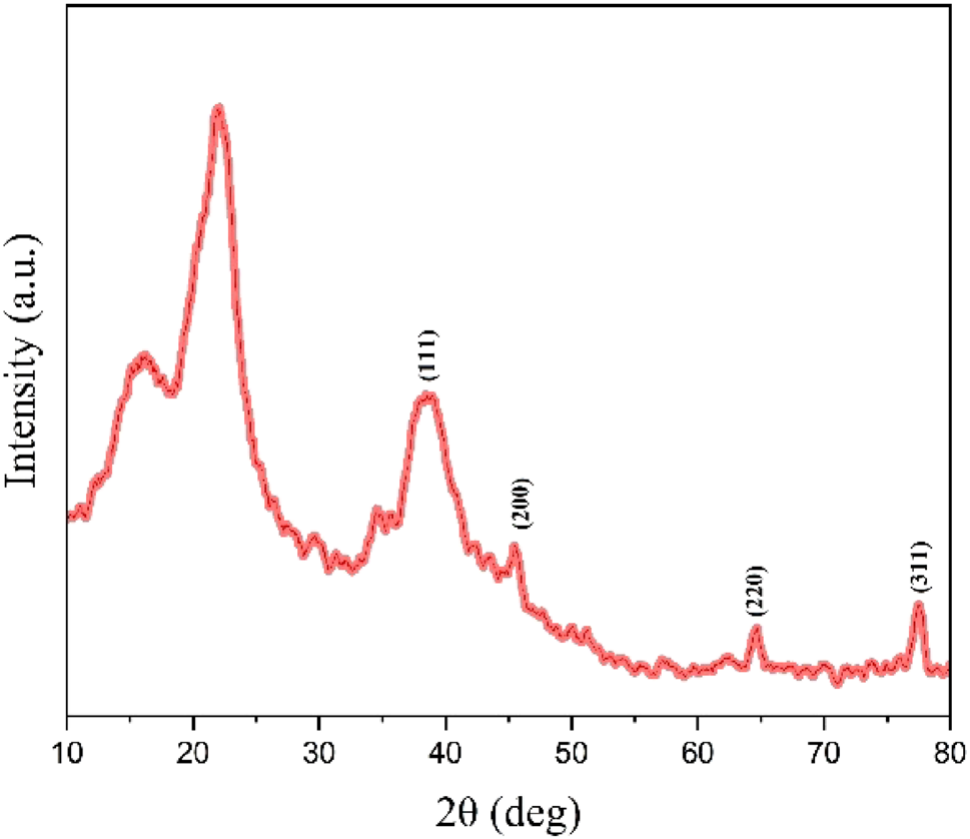
XRD pattern of LF/AgNPs.

The X-ray diffraction (XRD) pattern of LF/AgNPs is depicted in Fig. 7. The XRD pattern of LF/AgNPs displays peaks at 2θ of 35.84, 45.68, 64.76, and 77.64, which are the lattice planes (111), (200), (220), and (311), respectively, indicating the face-centered cubic (FCC) silver nanoparticle structure. Therefore, the XRD pattern clearly indicates that the synthesized silver nanoparticles are crystalline in nature^42^. Furthermore, the obtained diffraction patterns were compared with JCPDS card numbers, and it was found that they closely matched with the card number 04-0783.

**Figure 8:**
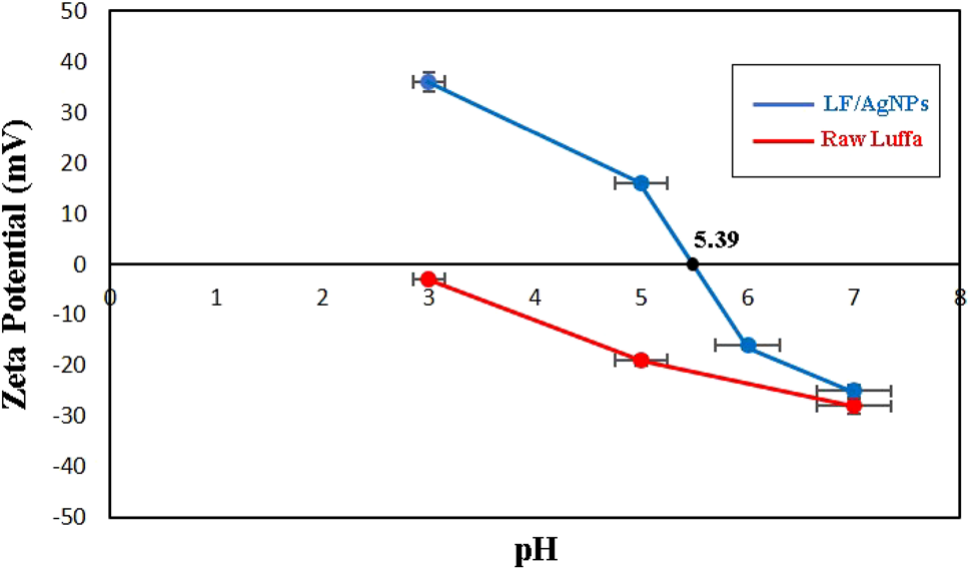
Diagrams of LF/AgNPs and raw luffa zeta potential at pH 3–7.

The potential zeta test was conducted to measure the surface charge of luffa and LF/AgNPs at pH 3–7. Figure 8 displays the outcomes, revealing that LF/AgNPs had a PZC value of 5.39. The original luffa material carried a negative charge, but the alteration of its surface with silver nanoparticles led to a modification of its charge. When the pH value is below 5.39, the LF/AgNPs material acquires a positive charge that is notably cationic when the pH is at 3. If the pH value exceeds 5.39, the surface of the adsorbent becomes negatively charged. It is worth noting that each particle demonstrates distinct characteristics at varying pH levels. By selecting the suitable pH level, this arrangement can govern the behavior and reaction of the adsorbents in different environments.

### Investigation of the Effect of various parameters on the adsorption process

#### Effect of agitation technique

**Figure 9:**
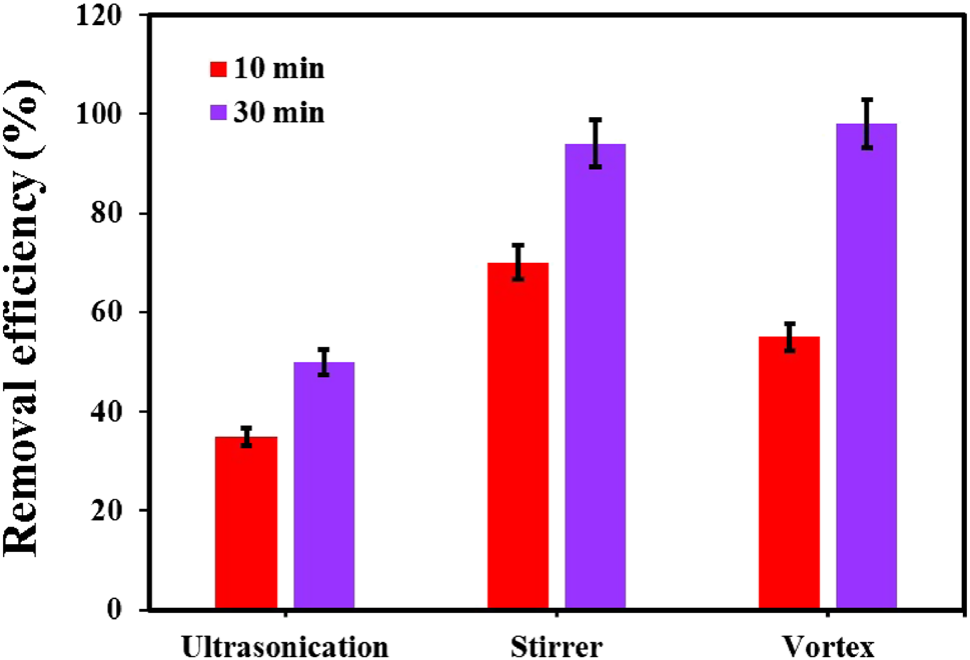
The effect of agitation technique within 10 and 30 min under the optimum conditions.

The stirring method is a crucial factor to consider as it enhances the contact area between the sample solution and the adsorbent. To this end, three stirring techniques, namely mechanical stirring, vortex, and ultrasonication, were assessed. As shown in Fig. 9, the elimination efficiency is greater for vortex and magnetic stirring. The reason for the lower removal rate in the ultrasonication method can be the expulsion of particles from the surface of the adsorbent due to the intensity of the ultrasonic waves.

#### Effect of solution pH

**Figure 10:**
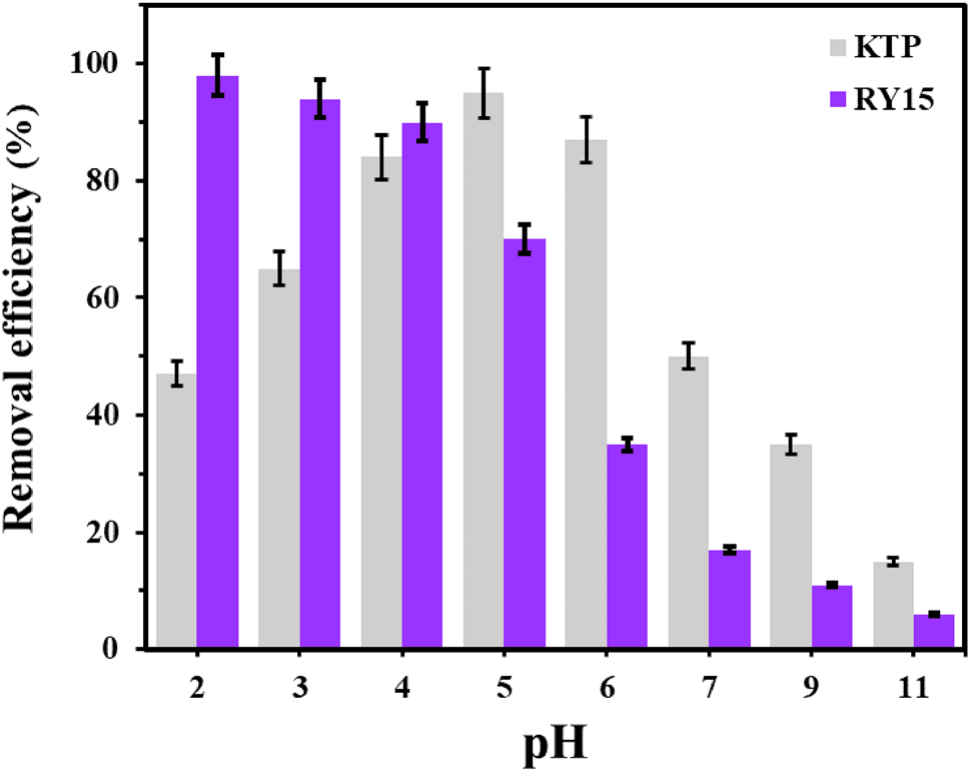
The effect of pH on the removal of RY15 and ketoprofen under the optimum conditions (contact times of 60 min and 40 min, adsorbent dosage of 25 mg and 25 mg, and initial concentrations of 100 mg/L for ketoprofen and 25 mg/L for RY15, respectively).

Another parameter that affects the adsorption process is pH. The initial pH value can be adjusted to determine the features of the adsorbent, such as the surface charge and ionization degree. In this study, the influence of pH was investigated over a range of 2–11. As previously mentioned, the LF/AgNPs had a PZC of 5.39. This implies that the surface charge is positive when the pH is less than 5.39, and negative when the pH is greater than 5.39. On the one hand, since ketoprofen is in the weak acidic range (ketoprofen pK_a_ = 4.45), it can exhibit different behaviors at different pH levels. For instance, at pH levels below pK_a_, ketoprofen exist as a neutral species, whereas at pH levels higher than pK_a_, it is present in anionic form. The pH-dependent adsorption of Ketoprofen onto LF/AgNPs is illustrated in Fig. 10. The peak adsorption rate was observed at pH = 5 for Ketoprofen, where the anionic form of ketoprofen can be electrostatically adsorbed onto the positively charged adsorbent. For pH values below pK_a_, the neutral form of Ketoprofen can adhere to the surface through Van der Waals interactions or hydrogen bonding. On the other hand, the removal efficiency of RY15 is also shown in Fig. 10. RY15 removal efficiency dropped from 98 to 6% by increasing the pH from 2 to 11. The higher rate of RY15 elimination by LF/AgNPs at acidic pH is assigned to the electrostatic attraction among the charged molecular substances of adsorbent and RY15 molecules. By reducing the pH to 2, these positively charged groups become more available. Electrostatic exchanges between these opposing groups with negative and positive charges could be the primary system concerning ketoprofen and RY15 removal. Through an electrostatic interaction, the negative ${-{\text{SO}}}_{3}^{-}$ group of the anionic dye interacts with a positive composite group, as reported in previous studies^65^.

#### Effect of contact time

**Figure 11:**
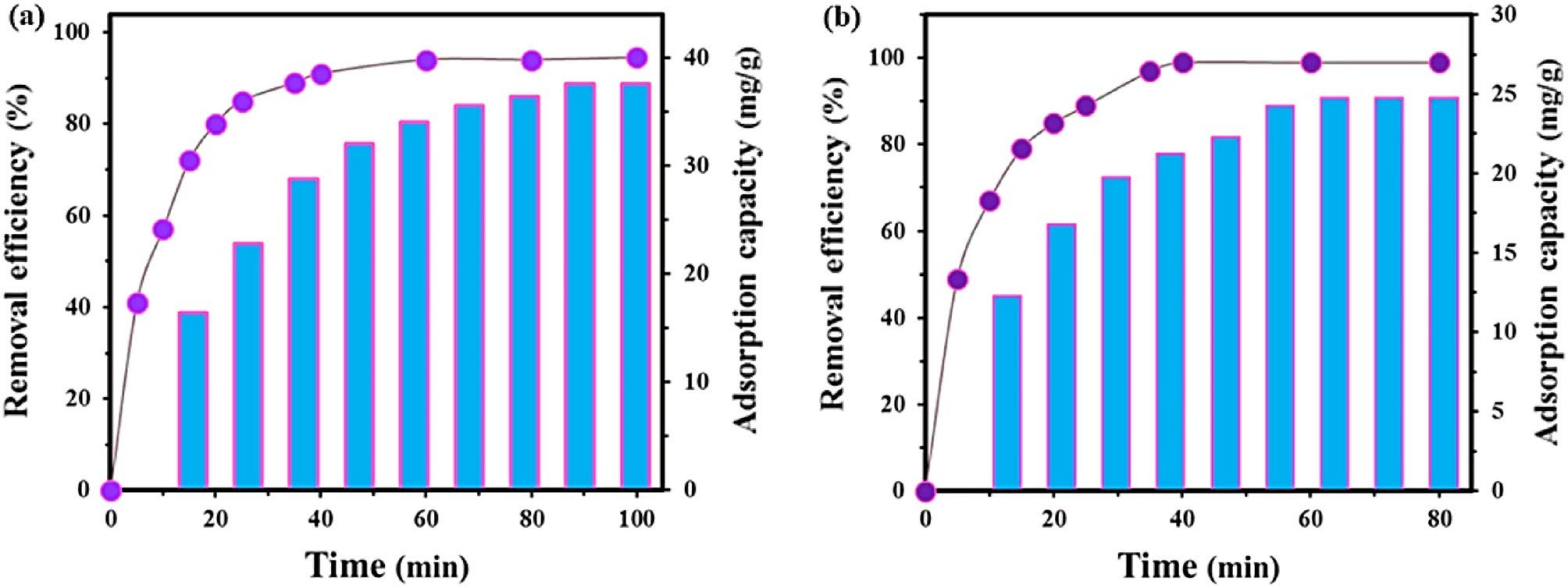
The effect of contact time on the (**a**) ketoprofen and (**b**) RY15 removal under the optimum conditions (pH levels of 5 and 2, adsorbent dosage of 25 mg and 25 mg, and initial concentrations of 100 mg/L for ketoprofen and 25 mg/L for RY15, respectively).

Typically, the elimination efficiency is dependent on the duration of contact between the adsorbate and sorbent. In general, the removal of pharmaceuticals tends to increase with the contact time until equilibrium is attained. After the equilibrium point, there is typically no significant further uptake of the adsorbate. The effect of contact time on the adsorption process of ketoprofen and RY15 were investigated in the range of 0–100 min for ketoprofen, and 0–80 min for RY15. As depicted in Fig. 11, the elimination rate of Ketoprofen and RY15 increased with an increase in time until a maximum time of 60 min and 40 min is reached respectively. The equilibrium state was achieved at 60 min for ketoprofen and 40 min for RY15. This suggests that the adsorbent becomes saturated with the adsorbate after the equilibrium point. Accordingly, at these points, the optimum contact times were established for the following investigations.

#### Impact of initial concentration

**Figure 12:**
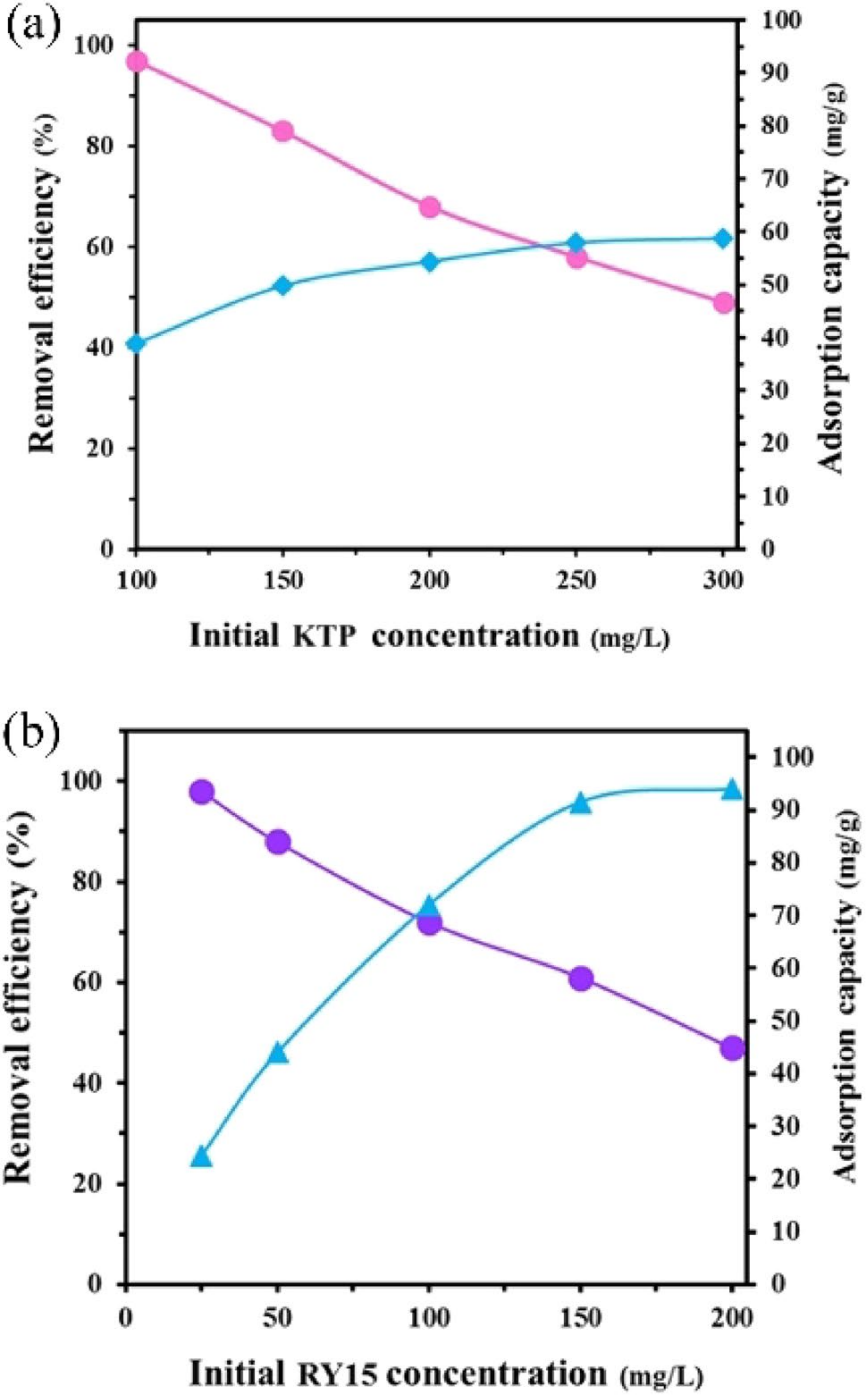
The effect of initial concentration on the (**a**) ketoprofen and (**b**) RY15 removal under the optimum conditions (pH levels of 5 and 2, contact times of 60 min and 40 min, and adsorbent dosage of 25 mg for ketoprofen and 25 mg for RY15, respectively).

The initial concentration is one of the most crucial factors that have an impact on the adsorption rate. It is necessary to confine the influence of the primary concentrations of ketoprofen and RY15. The effect of initial concentration on the removal efficiency is illustrated in Fig. 12a,b. The maximum removal efficiency of 98% was seen at both ketoprofen and RY15 in concentrations of 100 mg/L and 25 mg/L, respectively. The lowest removal of 49% for ketoprofen and 47% for RY15 was observed at 300 mg/L and 200 mg/L, respectively. At lower concentrations, the total available active zones remain constant, allowing effective removal of contaminants. As concentrations increase, the number of contaminant molecules rises, potentially exceeding the capacity of the available active zones. Consequently, higher concentrations result in lower removal efficiency, indicating that all active zones are not utilized efficiently. Just about all ketoprofen and RY15 were removed. Furthermore, the adsorption capacity is higher at lower concentrations because a greater proportion of available active sites can accommodate adsorbate molecules. At higher concentrations, the saturation of active sites may occur, leading to a plateau or decrease in adsorption capacity despite the overall increase in the number of adsorbate molecules in the system. To address this paradox and optimize factors, a concentration of 100 mg/L for ketoprofen and 25 mg/L for RY15 was selected for further investigations. This concentration optimization ensures effective utilization of available active zones while maintaining high removal efficiency for both ketoprofen and RY15.

#### Effect of adsorbent dosage

**Figure 13:**
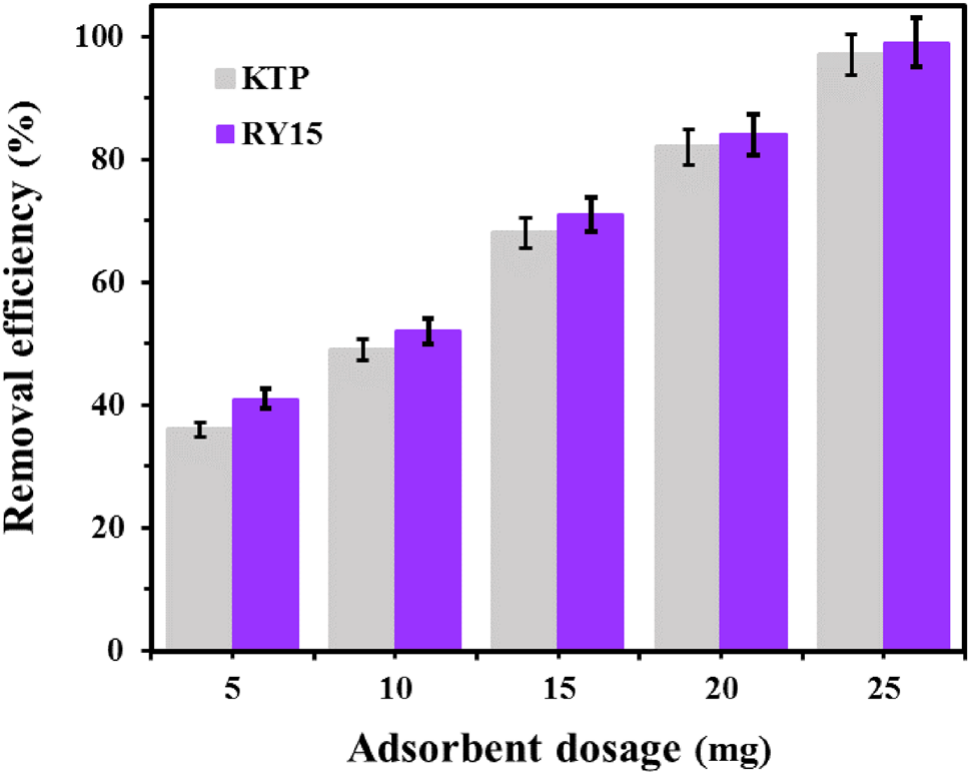
The effect of adsorbent dosage on the ketoprofen and RY15 removal under the optimum conditions (pH levels of 5 and 2, contact times of 60 min and 40 min, and initial concentrations of 100 mg/L for ketoprofen and 25 mg/L for RY15, respectively).

The significance of adsorbent dosage is primarily linked to the removal efficiency and overall cost of the process. The adsorption rate of Ketoprofen and RY15 were assessed by varying the dosage of LF/AgNPs at the optimal contact time. As depicted in Fig. 13, the highest removal rate of 97% and 99% was achieved for ketoprofen and RY15 respectively with a dosage of 25 mg of LF/AgNPs. As the amount of adsorbent increased, the removal percentage also increased, which is likely due to the increase in active sites resulting from the higher adsorbent dosage. A 25 mg sorbent dosage was chosen in the following investigations to optimize the remaining factors.

### Adsorption isotherm

Understanding the adsorption isotherm is important to determine an optimal elimination procedure. This study utilized the non-linear forms of Langmuir, Freundlich, Temkin, and Dubinin–Radushkevich (D–R) equations to characterize the equilibrium adsorption at a temperature of 25 °C. The constant factors related to the analyzed isotherms are described in Table 2. R^2^ (Regression coefficient values) defines the practicality of the model. The closer the R^2^ value goes to one, the better the isotherm model could define the elimination procedure. The Langmuir model is the most widely used equation that considers the adsorbed substance to form a monolayer with a thickness of one molecule. In the Langmuir model, the adsorption occurs as a single layer of molecules. The equation for the non-linear form of the Langmuir adsorption isotherm model is expressed as below^66,67^:

**Table 2:** Basic parameters calculated for isotherms.

Isotherm model	Parameters	Ketoprofen	Reactive yellow 15
Langmuir	q_m_ (mg/g)	56.882	97.766
	K_L_ (L/g)	0.669	0.149
	R^2^	0.904	0.912
Freundlich	K_F_ (mg/L)	34.869	29.734
	n	9.366	3.872
	R^2^	0.915	0.907
Temkin	b_T_ (J/mol)	476.541	179.766
	K_T_ (L/mg)	577.072	8.152
	R^2^	0.997	0.963
D-R	qm (mg/g)	55.392	87.527
	K (mol^2^/kJ^2^)	7.1E-07	4.88E-06
	R^2^	0.824	0.796

3 $${{\text{q}}}_{{\text{e}}}=\frac{{{\text{q}}}_{{\text{m}}}{{\text{K}}}_{{\text{L}}}{{\text{C}}}_{{\text{e}}}}{1+{{\text{K}}}_{{\text{L}}}{{\text{C}}}_{{\text{e}}}}$$

In the Langmuir adsorption isotherm model, q_e_ (mg/g) represents the equilibrium adsorption capacity, q_m_ (mg/g) is the maximum adsorption capacity, C_e_ (mg/L) is the equilibrium concentration of adsorbate in the solution after adsorption, and K_L_ (L/mg) is the Langmuir constant. R_L_ is an additional factor used to forecast the likelihood of the adsorption process. R_L_ is a unitless separation factor that should always fall between zero and one to indicate favorable adsorption (0 < R_L_ < 1). The isotherm type is determined by the value of R_L_, where R_L_ = 1 represents a linear isotherm, R_L_ > 1 indicates unfavorable adsorption, and R_L_ = 0 signifies irreversible adsorption. Equation (4) defines the separation factor R_L_, where C_0_ refers to the maximum initial concentration of the adsorbate, measures in parts per million (ppm), and K_L_ denotes the Langmuir constant^66–69^.

4 $${{\text{R}}}_{{\text{L}}}= \frac{1}{{{\text{C}}}_{0} {{\text{k}}}_{{\text{L}}}+1}$$

The Freundlich model operates on the premise that the adsorbent surface is non-uniform or heterogeneous in nature. This theory suggests that the active sites of the adsorbent are unevenly spread across its surface and possess varying energies, allowing for the adsorption of multiple layers. The Freundlich isotherm equation lacks a fundamental basis and is considered an empirical model. Equation (5) represents the Freundlich isotherm model^68,69^:

5 $${{\text{lnq}}}_{{\text{e}}}={{\text{lnk}}}_{\mathrm{F }}+ \frac{1}{{\text{n}}}\mathrm{ ln}{{\text{C}}}_{{\text{e}}}$$

Hence, if a graph is plotted with ln e on the horizontal axis and ln q_e_ on the vertical axis using linear regression, the surface adsorption data will adhere to the Freundlich isotherm model. In this situation, the intercept on the origin of the graph is equivalent to ln K_F_, while the slope is equal to 1/n. When 1/n = 0, it suggests the adsorption is irreversible; when 0 < 1/n < 1, it indicates a favorable adsorption process; and when 1/n > 1, it implies an unfavorable adsorption process. The Temkin isotherm, represented by Eq. 6, is used to determine the adsorption heat between Ketoprofen and RY15 onto the LF/AgNPs adsorbent. This isotherm considers the interaction between the two substances:

6 $${{\text{q}}}_{{\text{e}}}=(\frac{{\text{RT}}}{{{\text{b}}}_{{\text{T}}}}){\text{ln}}({{\text{K}}}_{\mathrm{T }}{{\text{C}}}_{{\text{e}}})$$

In the equation, R is the universal gas constant (8.314 J/mol K), ${{\text{b}}}_{{\text{T}}}$ (J/mol) is related to the adsorption heat constant, ${{\text{K}}}_{{\text{T}}}$ is the equilibrium binding constant (L/g), and T (K) is the temperature^69,70^. The Dubinin–Radushkevich isotherm model is a mathematical model used to describe the adsorption behavior of porous materials. The non-linear form of the D–R isotherm is particularly employed to analyze the adsorption of gases or solutes onto solid surfaces, often in the context of porous materials. Equation (7) represents the non-linear form of the D–R isotherm model:

7 $${{\text{q}}}_{{\text{e}}}={{\text{q}}}_{{\text{m}}}{\text{exp}}\left({-\mathrm{K\varepsilon }}^{2}\right)$$

8 $$\upvarepsilon ={\text{RTln}}(1+\frac{1}{{{\text{C}}}_{{\text{e}}}})$$

9 $${\text{E}}=\frac{1}{\sqrt{2{\text{K}}}}$$

qm (mg/g) is the maximum adsorption capacity, K (mol^2^/kJ^2^) is the D–R isotherm constant, ε (J/mol) is the Polanyi potential, given by Eq. 8, where R is the gas constant (8.314 J/mol K), T (K) is the temperature, and C_e_ (mg/L) is the equilibrium concentration of the adsorbate. Equation (9) is related to the calculation of the mean free energy using the Polanyi potential. The model assumes that the adsorption process occurs on a heterogeneous surface, and the Polanyi potential is used to account for the variation in adsorption energy on the surface. The non-linear D-R isotherm is valuable in understanding the adsorption mechanism and energetics in heterogeneous systems^66,67,71^.

**Figure 14:**
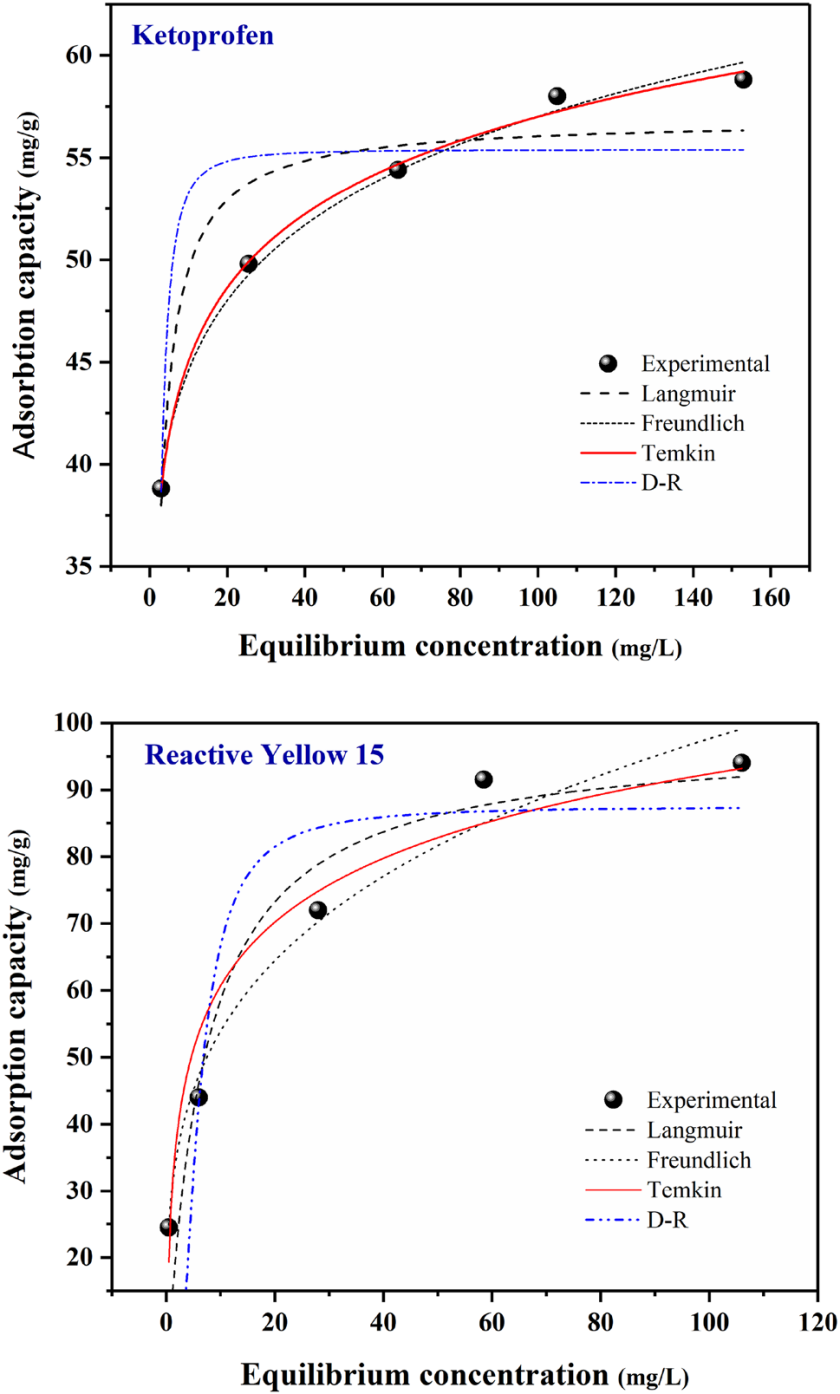
Isotherm fitted curves (non-linear) of Ketoprofen and RY15 adsorption.

Figure 14 illustrates a graphical representation of Langmuir, Freundlich, Temkin, and D–R isotherm models. Table 2 provides the calculated basic parameters for each of the isotherms. Although some regions of the surface exhibit single-layer adsorption, the Langmuir model does not fit the experimental data well, as indicated by its lower R^2^ values when compared to the Temkin model. In both scenarios involving the elimination of ketoprofen and RY15, the Temkin isotherm exhibits higher R^2^ values, specifically 0.997 for ketoprofen and 0.963 for RY15, demonstrating a strong correlation with the observed adsorption data. The Temkin isotherm model assumes a uniform spread of binding energies and accounts for the indirect interaction between LF/AgNPs and contaminants. According to the Temkin isotherm hypothesis, the adsorption heat decreases linearly with increasing surface coverage. The results affirm that the adsorption process of ketoprofen and RY15 can be characterized as a chemisorption process. The Temkin isotherm provides a means to quantify the heat of adsorption, where a positive b_T_ indicates an exothermic process. Furthermore, the D–R model was not fitted well to experimental data and has the lowest correlation coefficients for both contaminants.

### Adsorption kinetics

The rate of the adsorption procedure onto the LF/AgNPs adsorbent is significant in all interactions. The Pseudo first-order (PFO) kinetic equation is expressed as follows^4,72^:

10 $${{\text{q}}}_{{\text{t}}}={{\text{q}}}_{{\text{e}}}(1-{{\text{e}}}^{-{{\text{k}}}_{{\text{l}}}{\text{t}}})$$

11 $${\text{ln}}\left({{\text{q}}}_{{\text{e}}}-{{\text{q}}}_{{\text{t}}}\right)={{\text{lnq}}}_{{\text{e}}}-{{\text{K}}}_{{\text{l}}}{\text{T}}$$

K_1_ (1/min) represents the rate constant of the Pseudo first-order kinetic model. Another model used to describe the adsorption kinetics is the Pseudo second-order model (PSO), which can be expressed using the following equation:

12 $$\frac{{\text{t}}}{{{\text{q}}}_{{\text{t}}}}=\frac{{\text{t}}}{{{\text{q}}}_{{\text{e}}}}+\frac{1}{{{\text{k}}}_{2}{{\text{q}}}_{{\text{e}}}^{2}}$$

K_2_ (g/mg min) represents the rate constant of the Pseudo second-order kinetic model.

The third equation is the Elovich equation and is expressed as below:

13 $${{\text{q}}}_{{\text{t}}}=\frac{1}{\upbeta }{\text{ln}}(\mathrm{\alpha \beta t}+1)$$

$\mathrm{\alpha }$ is equivalent to the primary adsorption rate (mg/g min), and $\upbeta$ is the desorption constant (g/mg). Moreover, $\upbeta$ is linked to the surface coverage degree and activation energy for chemisorption.

Equation (14) represents the non-linear form of the fractional power equation:

14 $${{\text{q}}}_{{\text{t}}}={{\text{k}}}_{{\text{p}}}{{\text{t}}}^{{{\text{v}}}_{{\text{p}}}}$$

**Figure 15:**
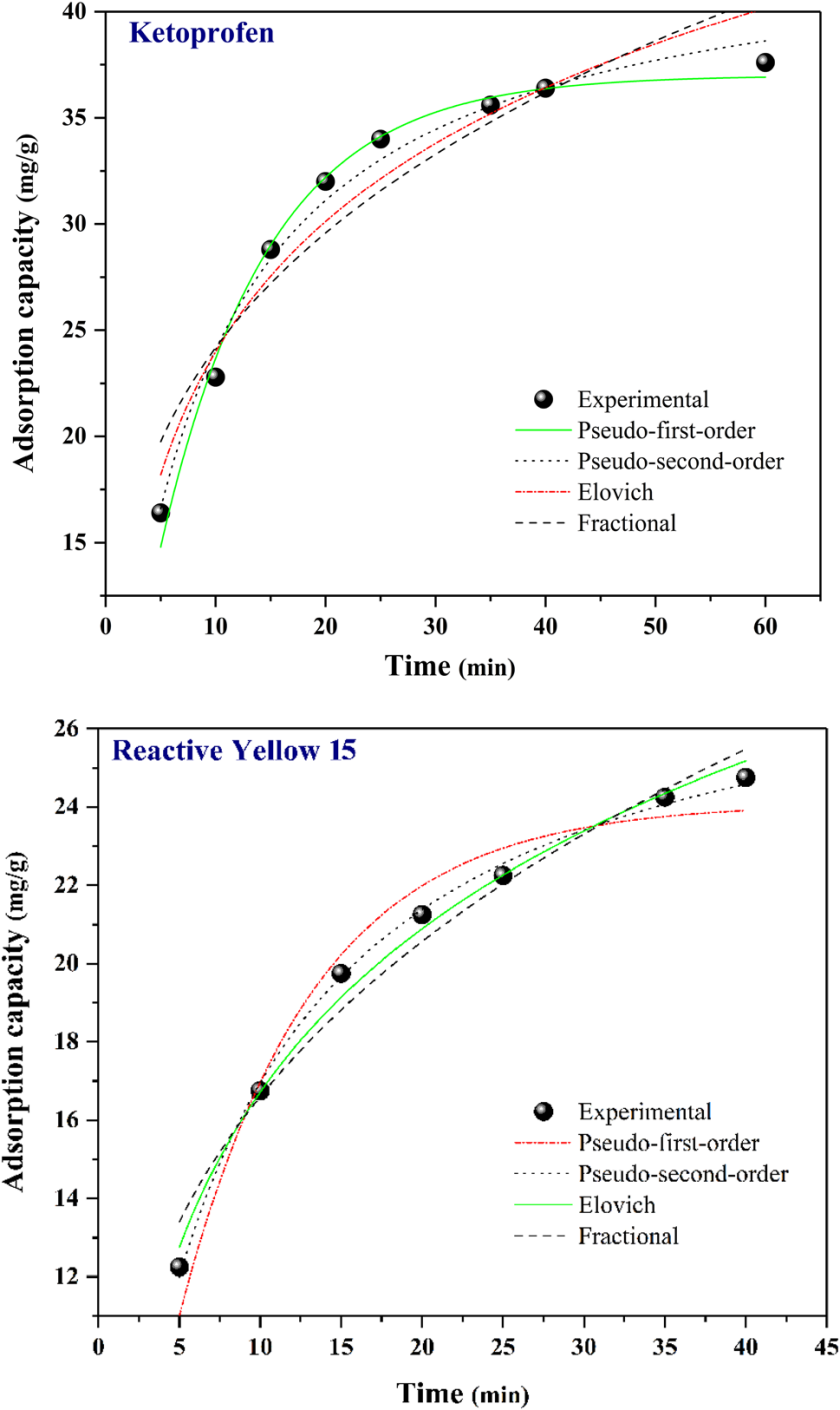
Kinetic fitted curves (non-linear) of ketoprofen and RY15 adsorption.

The antilogarithm of intercept directs to the ${{\text{k}}}_{{\text{p}}}$ value. ${q}_{t}$ is the amount of analyte adsorbed at time t. ${V}_{p}$ is a constant usually below one if adsorption kinetic data suits well into the power function model. Figure 15 illustrates the plots of Pseudo first-order, Pseudo second-order, Elovich, and fractional power adsorption kinetics^73,74^.

According to the data presented in Table 3, the correlation coefficient (R^2^) and adsorption capacity were evaluated to determine the most appropriate kinetic model. When comparing the correlation coefficients for ketoprofen removal, it was found that the pseudo-first-order model (R^2^ = 0.989) outperformed the other models indicating that it is the leading model that manages the adsorption procedure. The R^2^ values of pseudo-second-order, Elovich, and fractional power models were 0.987, 0.948, and 0.912 respectively. Regarding RY15 removal, the pseudo-second-order model demonstrated superior performance compared to other models, with a correlation coefficient (R^2^) of 0. 997. The R^2^ values of pseudo-first-order, Elovich, and fractional power models were 0.967, 0.992, and 0.972 respectively. Furthermore, when using the pseudo-second-order model, the calculated equilibrium capacity value of ketoprofen (q_e_, cal = 43.914 mg/g) and RY15(q_e_, cal = 28.925 mg/g), were found to be closer to the corresponding experimental value (q_e_, exp) compared to the results obtained from the other models. Figure 15 presents the results obtained from the mentioned models, including the plots generated through non-linear curve fitting. Table 3 presents the parameters associated with each of the adsorption kinetics.

**Table 3:** Kinetic parameters for RY15 and Ketoprofen adsorption.

Kinetic model	Parameters	Ketoprofen	Reactive Yellow 15
PFO	q_e_ (mg/g)	36.994	24.093
	K_1_(1/min)	0.098	0.122
	R^2^	0.989	0.967
PSO	q_e_ (mg/g)	43.914	28.925
	K_2_ (g/mg min)	0.002	0.005
	R^2^	0.987	0.997
Elovich	α (mg/g min)	11.167	8.169
	Β (g/mg)	0.106	0.157
	R^2^	0.948	0.992
Fractional	K_p_	12.364	8.148
	V_p_	0.291	0.309
	R^2^	0.912	0.972

## Possible mechanism of Ketoprofen and RY15 adsorption by LF/AgNPs adsorbents

**Figure 16:**
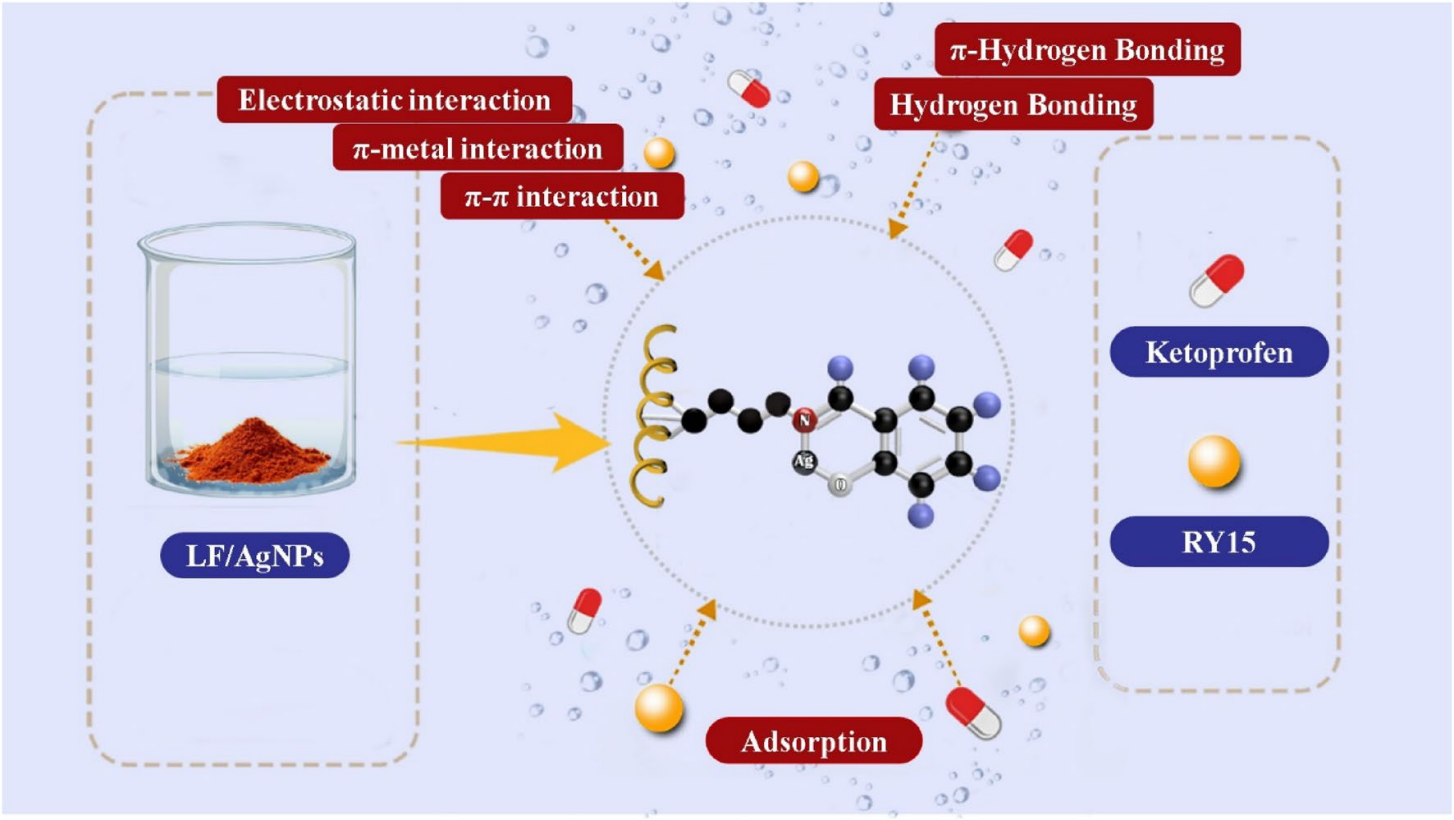
Possible mechanism of ketoprofen and RY15 adsorption on LF/AgNPs adsorbent.

Figure 16 presents the potential mechanism of Ketoprofen and RY15 adsorption by the LF/AgNPs. Through the modification of the surface of luffa with silver nanoparticles, it is hypothesized that various scenarios could occur. The best scenario for the adsorption mechanism of Ketoprofen and RY15 onto the LF/AgNPs involves the interaction of silver nanoparticles with the aromatic ring of Ketoprofen and RY15. The binding of Ag(I) to the conjugate system of the aromatic ring occurs through the π-metal bond^42,75,76^. Furthermore, the modification of the LF/AgNPs surface resulted in a positively charged adsorbent. This positive charge plays a crucial role in the adsorption of anionic Ketoprofen and RY15 in the. During the synthesis of the LF/AgNPs adsorbent, aromatic groups were introduced into the structure by adding salicylic aldehyde. These aromatic groups can form π–π interactions with the aromatic ring of Ketoprofen and RY15. Moreover, the hydrogen groups present on the surface of the luffa structure can result in the adsorption of Ketoprofen and RY15 molecules. Formation of hydrogen-bonding and π-Hydrogen bonding between the LF/AgNPs adsorbent and contaminants.

## Reusability of LF/AgNPs

**Figure 17:**
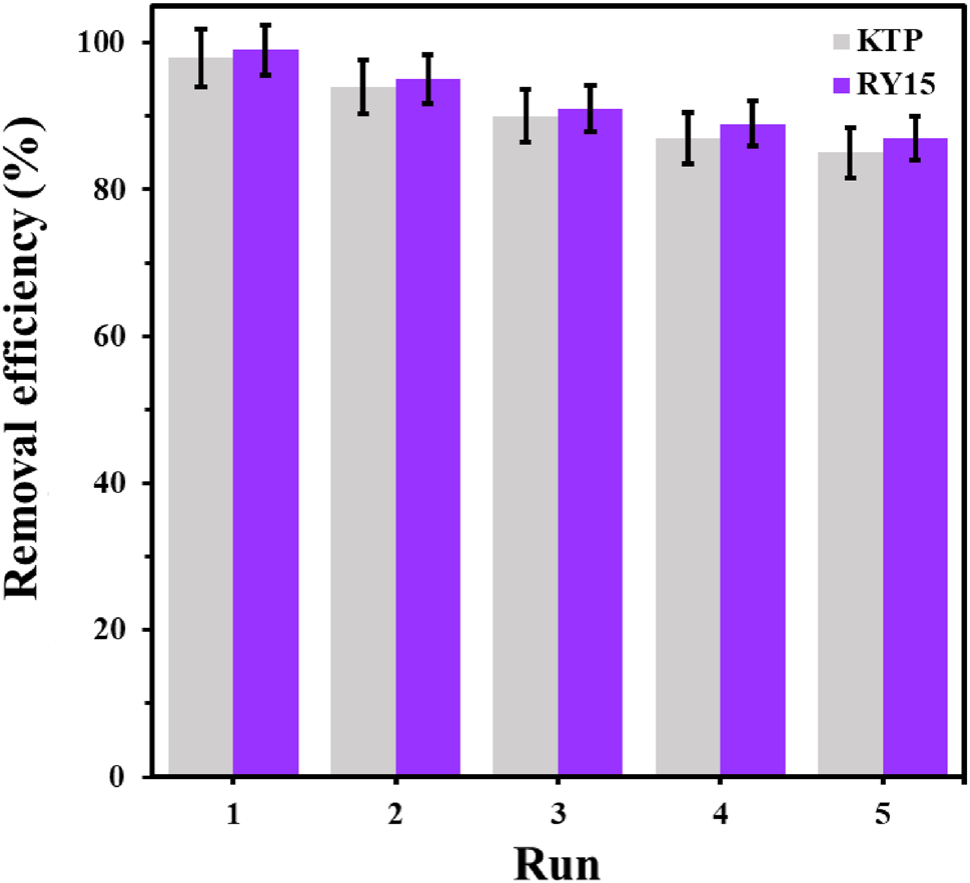
Reusability of LF/AgNPs for RY15 and ketoprofen adsorption.

From an economic standpoint, the choice of adsorbent holds paramount importance both in laboratory experiments and industrial applications in the field of adsorption. Researchers are actively exploring the utilization of economical biomaterials derived from natural sources that are abundantly available in the environment. The reusability of an adsorbent is one of the critical factors that affect the overall cost of the removal process^77^. The present study rigorously examined the reusability potential of LF/AgNPs under optimized conditions, as visually represented in Fig. 17. The results show that the removal percentage of Ketoprofen and RY15 decreased after undergoing five cycles of use. Impressively, even after experiencing five cycles of use, the removal efficiency for ketoprofen and RY15 adsorption consistently exceeded 85%. Washing LF/AgNPs with ethanol enhances the bonding of Ketoprofen and RY15 with the hydroxyl groups of ethanol, which facilitates the separation of the contaminants from the adsorbent surface. The reduced recovery process also suggests that some Ketoprofen and RY15 molecules tend to attach strongly to the adsorbent surface and are not easily separated during the recovery process. Therefore, in terms of reusability, LF/AgNPs adsorbents demonstrated satisfactory results for the adsorption of Ketoprofen and RY15.

## Comparison of LF/AgNPs with other adsorbents

Numerous studies have been conducted to eliminate Ketoprofen and RY15, emerging contaminants, from aqueous solutions. Tables 4 and 5 present a comparison of LF/AgNPs adsorbent with other natural-based adsorbents that have been investigated in recent years for their ability to remove Ketoprofen and RY15. Comparing various adsorbents based solely on their adsorption capacity is not sufficient. To make a more accurate comparison, other factors such as the removal percentage and contact time for adsorption should also be taken into consideration. LF/AgNPs adsorbent showed an excellent removal efficiency of 94% for ketoprofen, and 98% for RY15. The adsorption capacity for ketoprofen and RY15 were 56.88 and 97.76 mg/g respectively. In comparison to various natural-based adsorbents, the LF/AgNPs adsorbent demonstrates its suitability and practicality for effectively removing Ketoprofen and RY15 from aqueous solutions.

**Table 4:** Comparison with other adsorbents for the removal of Ketoprofen.

Adsorbents	Adsorption Capacity (mg/g)	Removal (%)	Contact Time (min)	References
Mesoporous silica SBA-15	0.28	86.5	120	^78^
molecularly imprinted polymer (MIP)	8.24	90	45	^79^
MnFe_2_O_4_/Bi_2_MoO_6_/PPy	22.21	87.03	120	^80^
Activated charcoal/H_3_PO_4_	24.7	88.4	120	^81^
Luffa/AgNPs	56.8	94	60	This study
NiFe_2_O_4_/activated carbon magnetic composite (NiAC)	97.75	85	240	^82^
Commercial carbon nanotubes	98.9	80	180	^83^
CSF@UiO-66	209.7	71.1	1200	^84^

**Table 5:** Comparison with other adsorbents for the removal of Reactive yellow 15.

Adsorbents	Adsorption Capacity (mg/g)	Removal (%)	Contact time (min)	References
Elephant Dung Activated Carbon	16.12	34.33	240	^85^
coconut coir activated carbon	31.90	99.3	30	^86^
Fe_3_O_4_/SiO_2_/ILNPs	63.69	90	20	^87^
Luffa/AgNPs	97.7	98	40	This study
Setaria verticillata	138.6	91	150	^88^

## Conclusion

This study has demonstrated that the LF/AgNPs adsorbent is highly effective in removing Ketoprofen and RY15 from water media. The addition of Ag nanoparticles to the surface of the adsorbent changes its charge from negative to positive, empowering electrostatic attraction with anionic Ketoprofen and RY15 and altogether progressing its adsorption proficiency. According to the results obtained, the maximum removal efficiencies of Ketoprofen and RY15 from aqueous solutions were 94% and 98% respectively, indicating the high performance of the LF/AgNPs adsorbent in removing Ketoprofen and RY15. The most likely mechanism for the adsorption of Ketoprofen and RY15 is the interaction between the π-electrons of the aromatic ring of Ketoprofen and RY15 and the existed metal (Ag) nanoparticles on the surface of the adsorbent. This type of interaction is known as π–π stacking, and it plays a significant role in the adsorption of organic compounds onto metal surfaces. When it comes to eliminating ketoprofen, the similarity in R^2^ values between the Freundlich and Temkin models implies that the adsorption process can adhere to both systems. This suggests the existence of multi-layer adsorption on the adsorbent's surface. In the case of RY15 removal, the Freundlich model is more suitable for describing the adsorption mechanism. Furthermore, it was observed that the pseudo-first-order model surpassed the other models in eliminating ketoprofen. This suggests that it is the primary model governing the adsorption process. In the case of RY15 removal, the pseudo-second-order model exhibited better performance in comparison to other models. Regarding reusability, it is noteworthy that the removal efficiency for ketoprofen and RY15 consistently stayed above 85% even after undergoing five usage cycles. Consequently, in the realm of recyclability, LF/AgNPs adsorbent demonstrated encouraging results for the adsorption of both Ketoprofen and RY15.

## Data Availability

All data generated or analyzed data for the experimental part of this study are included in this published article. The data that support the findings of this study are available from the corresponding author, [Afsaneh Mollahosseini], upon reasonable request. Moreover, all other data that support the plots within this paper and other findings of this study are available from the corresponding author upon reasonable request.

## References

[1] Brinza L, Maftei AE, Tascu S, Brinza F, Neamtu M (2022). Advanced removal of Reactive Yellow 84 azo dye using functionalised amorphous calcium carbonates as adsorbent. Sci. Rep..

[2] Rachna KM, Agarwal A, Singh NB (2019). Rice husk and Sodium hydroxide activated rice husk for removal of reactive yellow dye from water. Mater. Today Proc..

[3] Khadir A, Negarestani M, Ghiasinejad H (2020). Low-cost sisal fibers/polypyrrole/polyaniline biosorbent for sequestration of reactive orange 5 from aqueous solutions. J. Environ. Chem. Eng..

[4] Negarestani M (2023). Preparation of sisal fiber/polyaniline/bio-surfactant rhamnolipid-layered double hydroxide nanocomposite for water decolorization: kinetic, equilibrium, and thermodynamic studies. Sci. Rep..

[5] Sultana H, Usman M, Ul Haq A, Mansha A (2021). Micellar enhanced flocculation for the effective removal of reactive yellow 160 from synthetic textile effluent. Environ. Technol. Innov..

[6] Erol K, Uzun L (2017). Two-step polymerization approach for synthesis of macroporous surface ion-imprinted cryogels. J. Macromol. Sci. Part A.

[7] Kireç O, Alacabey İ, Erol K, Alkan H (2021). Removal of 17β-estradiol from aqueous systems with hydrophobic microspheres. J. Polym. Eng..

[8] Erol K, Yıldız E, Alacabey İ, Karabörk M, Uzun L (2019). Magnetic diatomite for pesticide removal from aqueous solution via hydrophobic interactions. Environ. Sci. Pollut. Res..

[9] Erol K, Gençer N, Arslan M, Arslan O (2013). Purification, characterization, and investigation of in vitro inhibition by metals of paraoxonase from different sheep breeds. Artif. Cells Nanomedicine, Biotechnol..

[10] Erol K, Teğin İ, Akdeniz S, Alacabey İ, Acar O (2023). Preconcentration and determination of Cu(II) and Cd(II) ions from wastewaters by using hazelnut shell biosorbent immobilized on Amberlite XAD-4 resin. MANAS J. Eng..

[11] Sultana M, Rownok MH, Sabrin M, Rahaman MH, Alam SMN (2022). A review on experimental chemically modified activated carbon to enhance dye and heavy metals adsorption. Clean. Eng. Technol..

[12] Hatimi B (2023). Physicochemical and statistical modeling of reactive Yellow 145 enhanced adsorption onto pyrrhotite Ash-Based novel (Catechin-PG-Fe)-Complex. Mater. Sci. Energy Technol..

[13] Tosun Satir İ, Erol K (2021). Calcined eggshell for removal of Victoria blue R dye from wastewater medium by adsorption. J. Turkish Chem. Soc. Sect. A Chem..

[14] Depci T, Alkan S, Kul A, Önal Y, Alacabey I, Dişli E (2011). “Characteristic properties of adsorbed catalase onto activated carbon based adiyaman lignite. Fresenius Environ. Bull..

[15] Rashtbari Y (2022). Green synthesis of zinc oxide nanoparticles loaded on activated carbon prepared from walnut peel extract for the removal of Eosin Y and Erythrosine B dyes from aqueous solution: experimental approaches, kinetics models, and thermodynamic studies. Environ. Sci. Pollut. Res..

[16] Rashtbari Y (2023). The optimization of reactive black 5 dye removal in the sono-catalytic process combined with local yellow montmorillonite and hydrogen peroxide using response surface methodology from aqueous solutions. Biomass Convers. Biorefinery.

[17] Pourali P, Behzad A, Ahmadfazeli A, Mokhtari SA, Rashtbari Y, Poureshgh Y (2022). Dissociation of acid blue 113 dye from aqueous solutions using activated persulfate by zero iron nanoparticle from green synthesis: the optimization process with RSM-BBD model: mineralization and reaction kinetic study. Biomass Convers. Biorefinery.

[18] Georgin J, Franco DSP, da Boit Martinello K, Lima EC, Silva LFO (2022). A review of the toxicology presence and removal of ketoprofen through adsorption technology. J. Environ. Chem. Eng..

[19] Negarestani M, Mollahosseini A, Farimaniraad H, Ghiasinejad H, Shayesteh H, Kheradmand A (2023). Efficient removal of non-steroidal anti-inflammatory ibuprofen by polypyrrole-functionalized magnetic zeolite from aqueous solution: kinetic, equilibrium, and thermodynamic studies. Sep. Sci. Technol..

[20] Khadir A, Negarestani M, Mollahosseini A (2020). Sequestration of a non-steroidal anti-inflammatory drug from aquatic media by lignocellulosic material (*Luffa cylindrica*) reinforced with polypyrrole: Study of parameters, kinetics, and equilibrium. J. Environ. Chem. Eng..

[21] Alacabey İ (2022). Antibiotic removal from the aquatic environment with activated carbon produced from pumpkin seeds. Molecules.

[22] Askari R (2023). Synthesis of activated carbon from walnut wood and magnetized with cobalt ferrite (CoFe_2_O_4_) and its application in removal of cephalexin from aqueous solutions. J. Dispers. Sci. Technol..

[23] Ahmadfazeli A, Poureshgh Y, Rashtbari Y, Akbari H, Pourali P, Adibzadeh A (2021). Removal of metronidazole antibiotic from aqueous solution by ammonia-modified activated carbon: adsorption isotherm and kinetic study. J. Water Sanit. Hyg. Dev..

[24] Shokoohia, R., Samadia, M. T., Amanib, M., Poureshgha, Y.: Optimizing laccase-mediated amoxicillin removal by the use of Box–Behnken design in an aqueous solution

[25] Shokoohi R, Samadi MT, Amani M, Poureshgh Y (2018). Modeling and optimization of removal of cefalexin from aquatic solutions by enzymatic oxidation using experimental design. Braz. J. Chem. Eng..

[26] Arana J, González S, Navarrete L, Caicedo O (2017). *Luffa cylindrica* as a natural adsorbent of cyanide ion in aqueous medium. Dyna.

[27] Anastopoulos I, Pashalidis I (2019). Τhe application of oxidized carbon derived from *Luffa cylindrica* for caffeine removal. Equilibrium, thermodynamic, kinetic and mechanistic analysis. J. Mol. Liq..

[28] Negarestani M, Lashkari A, Khadir A, Mollahosseini A, Muthu SS, Khadir A (2021). “Removal of rifampin by Luffa: A pharmaceutical potential in producing dye in water BT. Novel Materials for Dye-containing Wastewater Treatment.

[29] Mehrizad A, Aghaie M, Gharbani P, Dastmalchi S, Monajjemi M, Zare K (2012). Comparison of 4-chloro-2-nitrophenol adsorption on single-walled and multi-walled carbon nanotubes. Iranian J. Environ. Health Sci. Eng..

[30] Gharbani P, Mehrizad A, Jafarpour I (2015). Adsorption of penicillin by decaffeinated tea waste. Polish J. Chem. Technol..

[31] Mehrizad A (2017). Adsorption studies of some phenol derivatives onto Ag-cuttlebone nanobiocomposite: Modeling of process by response surface methodology. Res. Chem. Intermed..

[32] Erol K (2017). Polychelated cryogels: Hemoglobin adsorption from human blood. Artif. Cells Nanomed. Biotechnol..

[33] Erol K (2017). The adsorption of calmoduline via nicotinamide immobilized poly (HEMA-GMA) cryogels. J. Turk. Chem. Soc. Sect. A Chem..

[34] Erol K, Yavuz Ş (2022). Invertase adsorption with polymers functionalized by aspartic acid. J. Polym. Eng..

[35] Erol K, Bülter MB, Köse DA, Can HK (2021). Water-soluble polymeric particle embedded cryogels: Synthesis, characterisation and adsorption of haemoglobin. J. Polym. Eng..

[36] Feng Y (2018). Norfloxacin removal from aqueous solution using biochar derived from luffa sponge. J. Water Supply Res. Technol..

[37] da Costa JS, Fajardo AR (2023). Polypyrrole/stearic acid-coated Luffa cylindrica for enhanced removal of sodium diclofenac from water: Batch and continuous adsorption studies. J. Clean. Prod..

[38] Erol K (2016). DNA adsorption via Co(II) immobilized cryogels. J. Macromol. Sci. Part A.

[39] Li Z (2019). Efficient removal of heavy metal ions and organic dyes with cucurbit [8] uril-functionalized chitosan. J. Colloid Interface Sci..

[40] Motamedi M, Mollahosseini A, Negarestani M (2022). Ultrasonic-assisted batch operation for the adsorption of rifampin and reactive orange 5 onto engineered zeolite–polypyrrole/TiO_2_ nanocomposite. Int. J. Environ. Sci. Technol..

[41] Riza KA, Tolga D, Ihsan A, Salih A, Yunus O (2011). Equilibrium, kinetic and thermodynamic studies of nickel adsorption onto natural and modified kaolinites. Fresenius Environ. Bull..

[42] Joodaki S, Mollahosseini A (2023). Evaluation modified luffa with silver nanoparticles (LF/AgNPs) for removal of a nonsteroidal anti-inflammatory (IBUPROFEN) from aqueous media. Environ. Nanotechnol. Monit. Manag..

[43] Kheradmand A, Negarestani M, Mollahosseini A, Shayesteh H, Farimaniraad H (2022). Low-cost treated lignocellulosic biomass waste supported with FeCl_3_/Zn(NO_3_)_2_ for water decolorization. Sci. Rep..

[44] Rashtbari Y (2022). Potential of using green adsorbent of humic acid removal from aqueous solutions: Equilibrium, kinetics, thermodynamic and regeneration studies. Int. J. Environ. Anal. Chem..

[45] Erol K, Bolat M, Tatar D, Nigiz C, Köse DA (2020). Synthesis, characterization and antibacterial application of silver nanoparticle embedded composite cryogels. J. Mol. Struct..

[46] Erol K, Tatar D, Veyisoğlu A, Tokatlı A (2021). Antimicrobial magnetic poly(GMA) microparticles: Synthesis, characterization and lysozyme immobilization. J. Polym. Eng..

[47] Erol K (2017). Synthesis, characterization and chromatographic applications of antimicrobial cryogels TT—Antimikrobiyal Kriyojellerin Sentezi, Karakterizasyonu ve Kromatografik Uygulamaları. Hacettepe J. Biol. Chem..

[48] Rashtbari Y, Américo-Pinheiro JHP, Bahrami S, Fazlzadeh M, Arfaeinia H, Poureshgh Y (2020). Efficiency of zeolite coated with zero-valent iron nanoparticles for removal of humic acid from aqueous solutions. Water Air Soil Pollut..

[49] Vicente-Martínez Y, Caravaca M, Soto-Meca A, De Francisco-Ortiz O, Gimeno F (2020). Graphene oxide and graphene oxide functionalized with silver nanoparticles as adsorbents of phosphates in waters. A comparative study. Sci. Total Environ..

[50] Teğin İ, Demirel MF, Alacabey İ, Yabalak E (2022). Investigation of the effectiveness of waste nut shell–based hydrochars in water treatment: a model study for the adsorption of methylene blue. Biomass Convers. Biorefinery.

[51] Praipipat P, Ngamsurach P, Khamkhae P (2024). Iron(III) oxide-hydroxide modification on *Pterocarpus macrocarpus* sawdust beads for direct red 28 dye removal. Arab. J. Chem..

[52] Weng H (2023). Insight into FeOOH-mediated advanced oxidation processes for the treatment of organic polluted wastewater. Chem. Eng. J..

[53] Yu S-Y (2024). Review of advanced oxidation processes for treating hospital sewage to achieve decontamination and disinfection. Chin. Chem. Lett..

[54] Broda M (2020). Organosilicons of different molecular size and chemical structure as consolidants for waterlogged archaeological wood–a new reversible and retreatable method. Sci. Rep..

[55] Zeroual S (2020). Ethylene glycol based silver nanoparticles synthesized by polyol process: Characterization and thermophysical profile. J. Mol. Liq..

[56] Erol B, Erol K, Gökmeşe E (2019). The effect of the chelator characteristics on insulin adsorption in immobilized metal affinity chromatography. Process Biochem..

[57] Alacabey İ (2022). Endosulfan elimination using amine-modified magnetic diatomite as an adsorbent. Front. Chem..

[58] Ad C (2016). Kinetics, thermodynamics and equilibrium evaluation of adsorptive removal of iron from aqueous solution onto Algerian biosorbent’LUFFA CYLINDRICA’. J. Mater. Environ. Sci..

[59] Sodipo BK, Aziz AA (2014). A sonochemical approach to the direct surface functionalization of superparamagnetic iron oxide nanoparticles with (3-aminopropyl) triethoxysilane. Beilstein J. Nanotechnol..

[60] Pasternack RM, Rivillon Amy S, Chabal YJ (2008). Attachment of 3-(aminopropyl) triethoxysilane on silicon oxide surfaces: dependence on solution temperature. Langmuir.

[61] Zhou C, Liu H (2017). A novel nanofibrous film chemosensor for highly selective and sensitive optical signaling of Zn^2+^. J. Braz. Chem. Soc..

[62] Ardakani AA, Kargar H, Feizi N, Tahir MN (2018). Synthesis, characterization, crystal structures and antibacterial activities of some Schiff bases with N_2_O_2_ donor sets. J. Iran. Chem. Soc..

[63] Badineni V, Maseed H, Arla SK, Yerramala S, Naidu BVK, Kaviyarasu K (2021). Effect of PVA/PVP protective agent on the formation of silver nanoparticles and its photocatalytic and antimicrobial activity. Mater. Today Proc..

[64] Gharibshahi L, Saion E, Gharibshahi E, Shaari AH, Matori KA (2017). Structural and optical properties of Ag nanoparticles synthesized by thermal treatment method. Materials (Basel).

[65] Xu M-Y, Jiang H-L, Xie Z-W, Li Z-T, Xu D, He F-A (2020). Highly efficient selective adsorption of anionic dyes by modified β-cyclodextrin polymers. J. Taiwan Inst. Chem. Eng..

[66] Caliskan N, Kul AR, Alkan S, Sogut EG, Alacabey İ (2011). Adsorption of Zinc(II) on diatomite and manganese-oxide-modified diatomite: A kinetic and equilibrium study. J. Hazard. Mater..

[67] Sadeghalvad B, Khosravi S, Azadmehr AR (2016). Nonlinear isotherm and kinetics of adsorption of copper from aqueous solutions on bentonite. Russ. J. Phys. Chem. A.

[68] Singh RK, Kumar S, Kumar S, Kumar A (2008). Development of parthenium based activated carbon and its utilization for adsorptive removal of p-cresol from aqueous solution. J. Hazard. Mater..

[69] Garba ZN, Abdul Rahim A, Hamza SA (2014). Potential of *Borassus aethiopum* shells as precursor for activated carbon preparation by physico-chemical activation; optimization, equilibrium and kinetic studies. J. Environ. Chem. Eng..

[70] Mohammadi A, Khadir A, Tehrani RMA (2019). Optimization of nitrogen removal from an anaerobic digester effluent by electrocoagulation process. J. Environ. Chem. Eng..

[71] Alacabey İ (2022). Adsorptive removal of cationic dye from aqueous solutions using Bardakçı clay. Int. J. Agric. Environ. Food Sci..

[72] Negarestani M (2024). In-situ growth of Al/Ni layered double hydroxide onto polyaniline-wrapped sisal fibers for highly efficient removal of pharmaceutical Ketoprofen and Ibuprofen contaminants: Batch and fixed-bed column studies. J. Water Process Eng..

[73] Negarestani M, Farimaniraad H, Mollahosseini A, Kheradmand A, Shayesteh H (2023). Facile preparation of sisal–Fe/Zn layered double hydroxide bio-nanocomposites for the efficient removal of rifampin from aqueous solution: kinetic, equilibrium, and thermodynamic studies. Int. J. Phytoremediation.

[74] Negarestani M, Etemadifar P, Kheradmand A (2021). A mini-review on the application of magnetic-based MOF for dye elimination from polluted waters. Adv. Remov. Tech. Dye. Wastewaters.

[75] Khlobystov AN (2001). Supramolecular design of one-dimensional coordination polymers based on silver(I) complexes of aromatic nitrogen-donor ligands. Coord. Chem. Rev..

[76] Cuesta L, Sessler JL (2009). π-Metal complexes of tetrapyrrolic systems. A novel coordination mode in ‘porphyrin-like’ chemistry. Chem. Soc. Rev..

[77] Khadir A, Negarestani M, Azad A, Sillanpää M, Muthu SS, Khadir A (2021). The utilization of biomaterials for water purification: dyes, heavy metals, and pharmaceuticals. Novel Materials for Dye-containing Wastewater Treatment.

[78] Bui TX, Choi H (2009). Adsorptive removal of selected pharmaceuticals by mesoporous silica SBA-15. J. Hazard. Mater..

[79] Madikizela LM, Zunngu SS, Mlunguza NY, Tavengwa NT, Mdluli PS, Chimuka L (2018). Application of molecularly imprinted polymer designed for the selective extraction of ketoprofen from wastewater. Water SA.

[80] Wang Y, He L, Dang G, Li H, Li X (2021). Polypyrrole-functionalized magnetic Bi_2_MoO_6_ nanocomposites as a fast, efficient and reusable adsorbent for removal of ketoprofen and indomethacin from aqueous solution. J. Colloid Interface Sci..

[81] Baccar R, Sarrà M, Bouzid J, Feki M, Blánquez P (2012). Removal of pharmaceutical compounds by activated carbon prepared from agricultural by-product. Chem. Eng. J..

[82] Fröhlich AC, Foletto EL, Dotto GL (2019). Preparation and characterization of NiFe_2_O_4_/activated carbon composite as potential magnetic adsorbent for removal of ibuprofen and ketoprofen pharmaceuticals from aqueous solutions. J. Clean. Prod..

[83] Lawal IA, Lawal MM, Akpotu SO, Azeez MA, Ndungu P, Moodley B (2018). Theoretical and experimental adsorption studies of sulfamethoxazole and ketoprofen on synthesized ionic liquids modified CNTs. Ecotoxicol. Environ. Saf..

[84] Chen J (2022). Fabrication and adsorption mechanism of chitosan/Zr-MOF (UiO-66) composite foams for efficient removal of ketoprofen from aqueous solution. Chem. Eng. J..

[85] Theivarasu C, Chandra S (2013). Adsorption performance of activated carbon prepared from elephant (*Elephas maximus*) dung for the removal of reactive yellow 15 from aqueous solution. Desalin. Water Treat..

[86] Khan T, Kutty SRM, Chaudhuri M (2010). Adsorptive removal of reactive yellow 15 from aqueous solution by coconut coir activated carbon. Adsorpt. Sci. Technol..

[87] Golmohammadi F, Hazrati M, Safari M (2019). Removal of reactive yellow 15 from water sample using a magnetite nanoparticles coated with covalently immobilized dimethyl octadecyl[3-(trimethoxysilylpropyl)]ammonium chloride ionic liquid. Microchem. J..

[88] Vidhyadevi T (2014). Optimization of the process parameters for the removal of reactive yellow dye by the low cost *Setaria verticillata* carbon using response surface methodology: Thermodynamic, kinetic, and equilibrium studies. Environ. Prog. Sustain. Energy.

